# Combined Gastric Electrical Stimulation and Pyloroplasty in Gastroparesis: A Randomized Clinical Trial

**DOI:** 10.1001/jamanetworkopen.2025.46332

**Published:** 2025-12-09

**Authors:** Irene Sarosiek, Mohammad Bashashati, Brian R. Davis, Karina Espino, Denise Vasquez, Ryan Torelli, Tamis Bright, Jerzy Sarosiek, Jesus R. Diaz, Osvaldo Padilla, Alok K. Dwivedi, Richard W. McCallum

**Affiliations:** 1 Division of Gastroenterology, Department of Medicine, Texas Tech University Health Sciences Center, El Paso; 2 Division of Gastroenterology, Department of Medicine, Dell Medical School, The University of Texas at Austin; 3 Christus Trinity Clinic, Corpus Christi, Texas; 4 Paul L. Foster School of Medicine, Texas Tech University Health Sciences Center, El Paso; 5 Division of Endocrinology, Department of Medicine, Texas Tech University Health Sciences Center, El Paso; 6 Department of Radiology, Texas Tech University Health Sciences Center, El Paso; 7 Department of Pathology, Texas Tech University Health Sciences Center, El Paso; 8 Department of Biomedical Informatics, Biostatistics and Medical Epidemiology, University of Missouri School of Medicine, Columbia

## Abstract

**Importance:**

Patients with gastroparesis who do not respond to medical therapy may require surgical intervention, typically involving pyloroplasty (PP) alone or with implantation of a gastric electrical stimulation (GES) device.

**Objective:**

To investigate the outcomes of combining PP with GES in medication-refractory gastroparesis.

**Design, Setting, and Participants:**

This double-blind randomized clinical trial included patients who had diabetic or idiopathic gastroparesis. Patients from a US academic gastrointestinal motility clinic, who failed medical therapy, were included from January 10, 2017, to September 20, 2023. Patients were followed up for 6 months.

**Interventions:**

Patients with refractory gastroparesis underwent simultaneous implantation of GES with PP and were randomized into PP + GES-ON and PP + GES-OFF groups. In the PP + GES-ON group, the GES was turned on after surgery. In the PP + GES-OFF group, the device was kept off for 3 months and then was turned on for the following 3 months.

**Main Outcomes and Measures:**

Symptom scores measured with the Gastroparesis Cardinal Symptom Index (GCSI) and the total symptom score (TSS), gastric emptying, and hospitalization length of stay were recorded and compared at baseline and at 3-month and 6-month follow-up visits. Between-group comparisons at 3 months were performed using the Wilcoxon rank sum test following the intention-to-treat procedure.

**Results:**

The study included 38 patients with gastroparesis (24 females [63.2%]; mean [SD] age, 46.7 [13.2] years), of whom 31 (81.6%) had diabetic gastroparesis, and 7 (18.4%) had idiopathic gastroparesis. Patients were randomized to the PP + GES-ON (n = 19) or the PP + GES-OFF (n = 19) group. At 3 months, the improvement from baseline in the GCSI (median [IQR] ON: −2.2 [−2.6 to −1.5] vs median [IQR] OFF: −0.9 [−1.8 to −0.4]; median difference, −1.33 [95% CI, −2.34 to −0.33]; *P* = .01) and the TSS (median [IQR] ON: −15.0 [−16.0 to −8.0] vs median [IQR] OFF: −3.0 [−10.0 to −1.0]; median difference, −12.00 [95% CI, −17.49 to −6.51]; *P* = .005) was significantly greater in the PP + GES-ON compared with the PP + GES-OFF group. Both groups exhibited significantly faster and similar gastric emptying results compared with the baseline. When the PP + GES-OFF group had GES activated at 3 months, symptoms improved significantly by 6 months (median [IQR] GCSI at 6 months: 1.2 [0.4-2.5] vs at baseline: 3.3 [2.8-4.1]; median [IQR] TSS at 6 months: 8.0 [2.0-10.0] vs at baseline: 18 [14.0-21.0]), achieving results comparable with those patients who had their GES device on for the full 6 months. These results at 6 months were accompanied by a significant reduction in hospital length of stay (median [IQR] at 6 months: 0 [0-2.0] vs at baseline: 4.1 [0-10.1]) and an excellent safety profile.

**Conclusions and Relevance:**

In this randomized clinical trial, the combination of GES and PP yielded superior outcomes compared with PP alone, resulting in greater alleviation of gastroparesis symptoms and a reduction in hospitalization, which may enhance patient profiling and optimize decision-making for treatments.

**Trial Registration:**

ClinicalTrials.gov Identifier: NCT03123809

## Key Points

**Question:**

Does combining gastric electrical stimulation (GES) with pyloroplasty offer additional clinical benefits for patients with refractory gastroparesis?

**Findings:**

In this randomized clinical trial on combining pyloroplasty with GES in 38 adults with refractory gastroparesis, the improvement from baseline in gastroparesis symptom scores was significantly greater in the group with GES turned on after surgery compared with the group in which it was kept off for 3 months.

**Meaning:**

The findings of this study suggest that the combination of GES and pyloroplasty yields superior outcomes compared with pyloroplasty alone for refractory gastroparesis treatment by reducing symptoms safely and effectively.

## Teaser

This randomized clinical trial examines whether combining gastric electrical stimulation with pyloroplasty offers more clinical benefits for patients with refractory gastroparesis than treatment with pyloroplasty alone.

## Introduction

Gastroparesis is delayed gastric emptying (GE) without mechanical obstruction.^1^ In the US, its prevalence is up to 267.7 per 100 000 adult patients.^2^ Gastroparesis symptoms include nausea, vomiting, early satiety, bloating, and postprandial fullness.^3^ Some cases are idiopathic (IDGP), other cases are diabetic (DMGP), and some result from vagal nerve injury. Patients with gastroparesis often have nutritional deficits, reduced quality of life, poor diabetes control, frequent hospitalizations, and substantial economic burden.^1,4,5^ Treatment includes prokinetics, antiemetics, and enteral feeding. However, up to 40% of patients are refractory and may require gastric peroral endoscopic myotomy (G-POEM) or surgical interventions, such as gastric electrical stimulation (GES) or pyloroplasty (PP).^6,7,8^

GES is US Food and Drug Administration approved under a Humanitarian Device Exemption for medication-refractory IDGP and DMGP, offering about a 50% symptomatic response.^9^ However, it minimally affects GE, a crucial pathophysiological aspect of gastroparesis.^10^

GES has been approved as an implanted device for treating chronic nausea and vomiting associated with gastroparesis when pharmacologic treatments are insufficient.^6^ The device delivers low-energy, high-frequency electrical pulses to the stomach. GES is not a pacemaker but a neurostimulator that generates brief electrical impulses of low energy at frequencies of 12 per minute, higher than the physiologic 3 cycles per minute of the gastric slow wave. It does not reverse slow-wave dysrhythmia or change GE.^6,7^

Combining GES with PP has produced better improvements in clinical outcomes as opposed to PP alone,^11,12^ but this combination has never been investigated, to our knowledge, in a double-blind randomized clinical trial. We designed this randomized clinical trial to test whether concurrent GES and PP better alleviate symptoms and accelerate GE more than PP alone in refractory gastroparesis.

## Methods

### Study Design

This prospective, double-blind randomized clinical trial was conducted from January 10, 2017, to September 20, 2023, at the Gastrointestinal Motility Clinic, Texas Tech University Health Sciences Center, in El Paso, with the clinic’s institutional review board approval. Written informed consent was obtained from all participants. The trial protocol is presented in Supplement 1. The study followed the Consolidated Standards of Reporting Trials (CONSORT) reporting guideline.^13^

All patients underwent Heineke-Mikulicz PP with implantation of the GES system. Further details are in the eMethods in Supplement 2. PP involved a 3-cm to 4-cm longitudinal antro-pyloro-duodenal incision and transverse 2-layer closure. Intraoperative passage of a 10-mm to 12-mm gastroscope confirmed adequate PP. In some cases, an EndoFLIP (endoluminal functional lumen imaging probe) was used to assess pyloric compliance; a diameter more than 15 mm and distensibility more than 5 mm^2^/mm Hg indicated a successful PP.

Postoperatively, patients were randomized to either the PP + GES-ON or the PP + GES-OFF group. In the GES-ON group, the device was activated immediately; in the GES-OFF group (placebo), it remained inactive. At 3 months, GES was activated in the OFF group. Outcomes were assessed at 3 months and 6 months.

Randomization was performed by an unblinded physician (M.B.), who also activated GES before discharge. The surgeon (B.R.D.), outcome assessors (R.W.M. and coordinators), and patients were blinded. Block randomization was managed exclusively by the unblinded investigator (M.B.). Default GES parameters included a 330-μs pulse width, a 5-mA current, 14 Hz frequency, on time of 0.1 second, and off time of 5.0 seconds. Devices were not adjusted during the initial 3-month blinded phase (eMethods in Supplement 2).

### Enrollment Criteria

Eligible participants were adults (aged ≥18 years) with IDGP or DMGP for over 1 year, refractory to antiemetics and prokinetics, and had scintigraphic-confirmed delayed GE.^14^ Exclusion criteria included organic or pseudo-obstruction, primary eating or swallowing disorders, psychogenic vomiting, narcotic dependency, morbid obesity, and active malignant neoplasms.

### Clinical Outcome Assessment

Primary outcomes were the total symptom score (TSS) and the Gastroparesis Cardinal Symptom Index (GCSI); greater score reductions indicated improvement.^15^ A decrease of 5 or more points in the TSS and 1 or more points in the GCSI, with Cohen *d* greater than or equal to 0.8, was defined as the minimal clinically important difference. Secondary outcomes included the Patient Assessment of Gastrointestinal Disorders Symptom Severity Index (PAGI-SYM),^15^ GE, hemoglobin A_1c_ (HbA_1c_) in DMGP, and hospital length of stay (HLOS) (eMethods in Supplement 2). Outcomes were assessed at baseline, 3 months, and 6 months. GE was reassessed at 3 months. HLOS, due to gastroparesis-related symptoms or complications, was recorded at baseline, 0 to 3 months, and 3 to 6 months after surgery. Baseline HLOS was calculated from hospitalizations within 12 months before surgery and averaged over a 3-month period. Hospitalizations related to PP and GES implantation were excluded.

### Sample Size

The sample size was calculated to assess the efficacy of PP + GES-ON compared with PP + GES-OFF on TSS and GCSI measures. A previous study reported an improvement in mean (SE) GCSI scores in the PP + GES group (1.1 [0.2]) compared with PP or GES alone (0.9 [0.2]).^11^ We hypothesized that combined GES and PP would produce a similar effect size (Cohen *d* = 1.0) for the TSS and the GCSI compared with PP alone. To detect a significant difference with 80% power and a 5% α level of significance using a 2-sided unpaired *t* test or Wilcoxon rank sum test, it was calculated that at least 18 patients per group were required. To account for a 20% dropout, 22 patients per group were enrolled. No interim analysis was planned. Further information is provided in the eMethods in Supplement 2.

### Statistical Analysis

The primary outcome analysis followed an intention-to-treat approach, followed by analyses excluding patients who were deceased or lost to follow-up. Post hoc power calculations assessed the impact of exclusions on effect size. Quantitative data were summarized using mean (SD) and/or medians and IQRs (quartile 1, quartile 3); categorical variables were presented as frequencies (percentages). Between-group comparisons used unpaired *t* tests, Wilcoxon rank sum, or χ^2^ tests as appropriate. Changes in outcomes at 3 months and 6 months were compared using Wilcoxon rank sum tests and validated with unpaired *t* tests. Median regression between groups was used to estimate effect sizes and 95% CIs. Regression was also used to adjust for baseline covariates. Within-group changes were analyzed with Wilcoxon signed rank tests and validated by repeated-measures analysis of variance (ANOVA) with Bonferroni correction. Due to multiple coprimary outcomes, significance was set at a 2-sided α = .01. Statistical analyses were performed using Stata, version 17 (StataCorp LLC).^16^

## Results

Data from 38 patients with refractory gastroparesis (24 females [63.2%] and 14 males [36.8%]; mean [SD] age, 46.7 [13.2] years), who were randomized to PP + GES-ON (n = 19) or PP + GES-OFF (n = 19) groups, were analyzed. Among the patients, 31 (81.6%) had DMGP, and 7 (18.4%) had IDGP. Five patients with gastroparesis (13.2%) underwent laparotomy due to the history of previous abdominal surgeries and potential adhesions, of whom 4 were randomized to the ON group and 1 to the OFF group. None of the laparoscopic procedures were converted to open surgery. 

**Figure 1.**
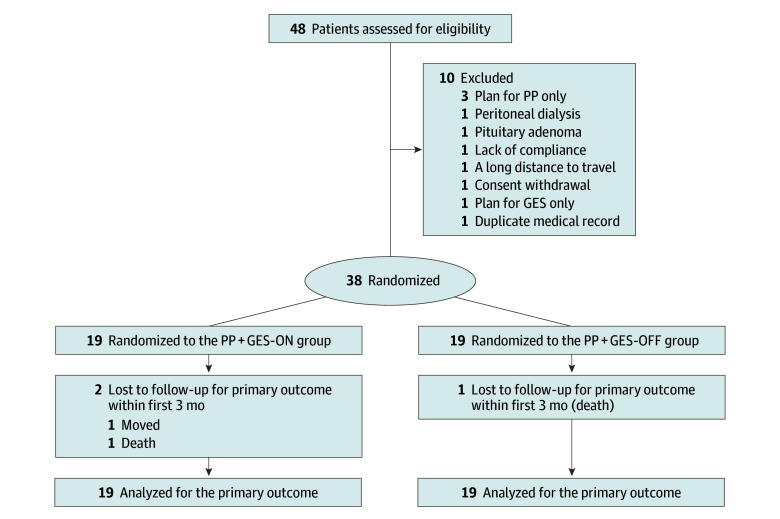
Patient Flow Diagram Patient recruitment and study process to compare the pyloroplasty with gastric electrical stimulation (PP + GES)-ON vs PP + GES-OFF groups. In the GES-ON group, the device was activated immediately; in the GES-OFF group, it remained inactive until 3 months after surgery.

The study design, patient flow, and allocation to the PP + GES-ON and PP + GES-OFF groups are presented in Figure 1. Baseline clinical characteristics were comparable between the PP + GES-ON and PP + GES-OFF groups (Table 1).

**Table 1.** Baseline Characteristics Between Randomized Intervention Groups

Characteristic	Group, No. (%)^a^	
	PP + GES-ON (n = 19)	PP + GES-OFF (n = 19)
Sex		
Female	14 (74)	10 (53)
Male	5 (26)	9 (47)
Gastroparesis		
Diabetic	14 (74)	17 (89)
Idiopathic	5 (26)	2 (11)
Age, mean (SD), y	47.1 (12.2)	46.4 (14.4)
Duration of gastroparesis, median (IQR), y	4.0 (2.0-8.0)	3.0 (2.0-4.0)
Body mass index, median (IQR)^b^	25.4 (21.6-27.5)	24.8 (23.5-31.6)
Score, median (IQR)		
Total symptom score^c^		
Vomiting	3.0 (3.0-4.0)	3.0 (3.0-4.0)
Nausea	4.0 (3.0-4.0)	3.5 (3.0-4.0)
Early satiety	3.0 (3.0-4.0)	3.0 (3.0-4.0)
Bloating	3.0 (2.0-3.0)	3.0 (2.0-4.0)
Postprandial fullness	3.0 (3.0-4.0)	3.0 (2.0-4.0)
Epigastric pain	3.0 (3.0-4.0)	3.0 (2.0-4.0)
Total	20.0 (18.0-22.0)	18.0 (14.0-21.0)
Gastroparesis Cardinal Symptom Index^d^		
Total	3.8 (3.2-4.4)	3.3 (2.8-4.1)
Nausea or vomiting	4.0 (3.0-4.7)	3.8 (3.3-4.7)
Postprandial fullness	3.9 (3.5-4.5)	3.6 (2.3-4.3)
Bloating	3.5 (3.0-4.0)	3.8 (1.5-5.0)
PAGI-SYM^e^	3.6 (2.8-4.1)	2.9 (2.1-3.8)
Hemoglobin A_1c_ in DMGP, median (IQR)	7.6 (6.9-10.0)	8.8 (7.3-10.7)
Hospitalization, median (IQR), d	0.4 (0-11.2)	4.1 (0-10.1)
Presence of hospitalizations	11 (58)	14 (74)
Gastric emptying retention, median (IQR), %		
At 2 h	84.0 (65.0-94.0)	69.0 (60.0-80.0)
At 4 h	45.5 (31.6-60.0)	38.5 (27.0-57.0)

Abbreviations: DMGP, diabetic gastroparesis; GES, gastric electrical stimulation; PAGI-SYM, Patient Assessment of Upper Gastrointestinal Disorders Symptom Severity Index; PP, pyloroplasty.

^a^ In the GES-ON group, the device was activated immediately; in the GES-OFF group, it remained inactive until 3 months after surgery.

^b^ Calculated as weight in kilograms divided by height in meters squared.

^c^ Scores range from 0 to 24, with higher scores indicating more severe symptoms.

^d^ Scores range from 0 to 5, with higher scores indicating more severe symptoms.

^e^ Scores range from 0 to 5, with higher scores indicating more severe symptoms.

### Effects of the 2 Groups on Outcomes at the 3-Month Visit

**Figure 2.**
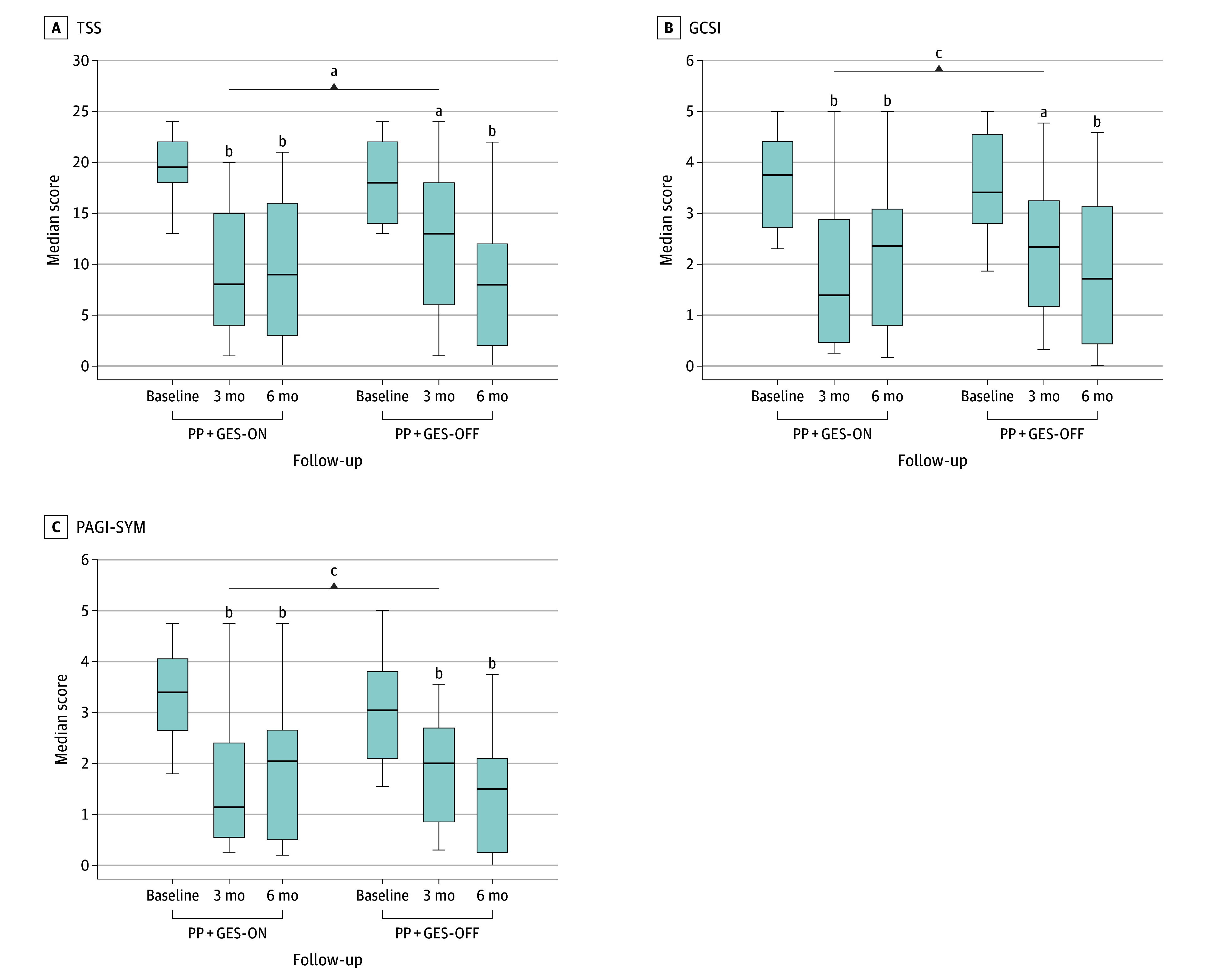
Total Symptom Score (TSS), Gastroparesis Cardinal Symptom Index (GCSI), and Patient Assessment of Upper Gastrointestinal Disorders Symptom Severity Index (PAGI-SYM) Results Representing the Pyloroplasty With Gastric Electrical Stimulation (PP + GES)-ON and PP + GES-OFF Groups In the GES-ON group, the device was activated immediately. At 3 months, the median TSS (A), GCSI (B), and PAGI-SYM (C) results were significantly lower in the PP + GES-ON group compared with the PP + GES-OFF group. At this time, the GES was activated in the PP + GES-OFF group; thus both groups were considered ON groups by the 6-month follow-up. In the TSS, scores range from 0 to 24, with higher scores indicating more severe symptoms. In the GCSI, scores range from 0 to 5, with higher scores indicating more severe symptoms. In the PAGI-SYM, scores range from 0 to 5, with higher scores indicating more severe symptoms. Horizontal lines inside boxes indicate medians; outer horizontal lines, IQRs; whiskers, ranges. ^a^*P* < .01. ^b^*P* < .001. ^c^*P* < .05.

At the 3-month follow-up, the PP + GES-ON group demonstrated a significantly greater median (IQR) change in improvement in the TSS (−15.0 [−16.0 to −8.0]) compared with the PP + GES-OFF (−3.0 [−10.0 to −1.0]) group (median difference, −12.00 [95% CI, −17.49 to −6.51]; *P* = .005). As with the TSS, the median (IQR) change in improvement in the GCSI (ON: −2.2 [−2.6 to −1.5] vs OFF: −0.9 [−1.8 to −0.4]; median difference, −1.33 [95% CI, −2.34 to −0.33]; *P* = .01) and the PAGI-SYM (ON: −2.1 [−2.8 to −1.3] vs OFF: −0.8 [−1.6 to −0.1]; median difference, −1.25 [95% CI, −2.17 to −0.33]; *P* = .009) also yielded greater improvements in the PP + GES-ON group compared with the PP + GES-OFF group. Effect sizes for the main outcomes were calculated as Cohen *d* = 1.7 for the TSS and Cohen *d* = 1.1 for the GCSI. Vomiting and epigastric pain scores as components of the TSS and of the GCSI nausea or vomiting domain subscale showed statistically significant improvement in the PP + GES-ON group compared with the PP + GES-OFF group (Figure 2 and eTables 1 and 2 and eFigure in Supplement 2). These results were unchanged in adjusted regression analyses as well (eTable 2 in Supplement 2). However, no significant differences were observed in HLOS and GE results, suggesting that PP alone sufficed to accelerate GE, and GES did not further accelerate emptying. These findings were mostly consistent in parametric data analysis after removing deaths and loss to follow-up data as well (eTable 3 in Supplement 2).

### Effects of PP + GES-ON Group on Outcomes at 3 and 6 Months After Surgery

In the PP + GES-ON group, the median (IQR) symptom scores including the TSS (7.0 [4.0-11.0] vs 20.0 [18.0-22.0]; *P* < .001), the GCSI (1.3 [0.5-2.7] vs 3.8 [3.2-4.4]; *P* < .001), the PAGI-SYM (1.1 [0.6-2.3] vs 3.6 [2.8-4.1]; *P* < .001), HLOS (0 vs 0.4 [0-11.3]; *P* = .05), and HbA_1c_ in DMGP (6.9 [5.5-7.5] vs 7.6 [6.9-10.0]; *P* = .05) were found to be significantly reduced at the 3-month follow-up compared with their respective baseline values. The improvements in symptom scores remained significant at the 6-month follow-up compared with their respective baseline values (Table 2). In addition to symptom scores, a significant median (IQR) improvement in GE retention at 2 hours (49.5% [33.5%-80.0%] vs 84.0% [68.0%-94.0%]; *P* = .01) and at 4 hours (12.5% [6.0%-26.0%] vs 45.5% [31.0%-60.0%]; *P* = .01) was noticed compared with the baseline values. However, no significant changes in any outcome were observed in 6 months compared with 3 months. These findings were unchanged in parametric data analysis using repeated-measures ANOVA followed by Bonferroni correction (eTable 4 in Supplement 2).

**Table 2.** Outcome Measures in the PP + GES-ON Group at 3 Months and 6 Months After Surgery^a^

Factor	Median (IQR)			*P* value^b^		
	Baseline	3 mo	6 mo	Baseline vs 3 mo	Baseline vs 6 mo	3 mo vs 6 mo
Total symptom score^c^						
Total	20.0 (18.0-22.0)	7.0 (4.0-11.0)	8.0 (3.0-14.0)	<.001^d^	<.001^d^	>.99
Vomiting	3.0 (3.0-4.0)	1.0 (0-2.0)	2.0 (0-2.0)	<.001^d^	.008^d^	.98
Nausea	4.0 (3.0-4.0)	2.0 (1.0-2.0)	1.0 (1.0-2.0)	<.001^d^	.001^d^	>.99
Early satiety	3.0 (3.0-4.0)	2.0 (0-2.0)	2.0 (1.0-3.0)	.002^d^	.05^d^	.14
Bloating	3.0 (2.0-3.0)	1.0 (0-2.0)	1.0 (0-3.0)	.001^d^	.005^d^	>.99
Postprandial fullness	3.0 (3.0-4.0)	2.0 (1.0-2.0)	2.0 (1.0-3.0)	.002^d^	.002^d^	.81
Epigastric pain	3.0 (3.0-4.0)	1.0 (0-1.0)	1.0 (0-2.0)	<.001^d^	.003^d^	>.99
Gastroparesis Cardinal Symptom Index^e^						
Total	3.8 (3.2-4.4)	1.3 (0.5-2.7)	2.3 (0.7-2.9)	<.001^d^	<.001^d^	.68
Nausea or vomiting	4.0 (3.0-4.7)	1.0 (0.3-2.0)	2.3 (0.3-2.8)	<.001^d^	.002^d^	.44
Postprandial fullness	3.9 (3.5-4.5)	1.5 (1.0-2.8)	1.9 (1.1-3.1)	<.001^d^	.003^d^	>.99
Bloating	3.5 (3.0-4.0)	0.5 (0-2.5)	1.0 (0.3-2.3)	.003^d^	.001^d^	>.99
PAGI-SYM^f^	3.6 (2.8-4.1)	1.1 (0.6-2.3)	1.7 (0.4-2.3)	<.001^d^	<.001^d^	>.99
Hemoglobin A_1c_ in DMGP	7.6 (6.9-10.0)	6.9 (5.5-7.5)	6.3 (5.0-8.4)	.05	.75	>.99
Hospitalization, d	0.4 (0-11.3)	0	0	.05	.56	>.99
Presence of hospitalizations, No. (%)	10 (56)	4 (24)	2 (22)	.08	.24	>.99
Gastric emptying retention, %						
At 2 h	84.0 (68.0-94.0)	49.5 (33.5-80.0)	NA	.01^d^	NA	NA
At 4 h	45.5 (31.0-60.0)	12.5 (6.0-26.0)	NA	.01^d^	NA	NA

Abbreviations: DMGP, diabetic gastroparesis; GES, gastric electrical stimulation; NA, not applicable; PAGI-SYM, Patient Assessment of Upper Gastrointestinal Disorders Symptom Severity Index; PP, pyloroplasty.

^a^ In the GES-ON group, the device was activated immediately. Nonparametric Friedman test analysis was followed by the Wilcoxon signed rank test after adjusting for Bonferroni correction for multiple comparisons.

^b^ Calculated using the Wilcoxon signed rank test except for hospitalization using McNemar tests.

^c^ Scores range from 0 to 24, with higher scores indicating more severe symptoms.

^d^ Result remained significant at 1% even after correcting for multiplicity due to multiple outcomes.

^e^ Scores range from 0 to 5, with higher scores indicating more severe symptoms.

^f^ Scores range from 0 to 5, with higher scores indicating more severe symptoms.

### Effects of PP + GES-OFF Group on Outcomes at 3 and 6 Months After Surgery

The median (IQR) symptom scores including the TSS (12 [6.0-18.0] vs 18 [14.0-21.0]; *P* = .006), the GCSI (2.3 [1.2-3.1] vs 3.3 [2.8-4.1]; *P* = .002), and the PAGI-SYM (1.8 [0.9-2.7] vs 2.9 [2.1-3.8]; *P* < .001) were found to be significantly reduced at the 3-month follow-up after PP alone (PP + GES-OFF) compared with their respective baseline values, without significant changes in HLOS and HbA_1c_ in DMGP. In addition to symptom scores, a significant median (IQR) improvement in GE retention at 2 hours (38.0% [28.0%-48.0%] vs 69.0% [60.0%-80.0%]; *P* < .001) and at 4 hours (10.0% [7.0%-25.0%] vs 38.5% [27.0%-57.0%]; *P* = .002) was noticed after PP alone compared with the baseline values. After activating GES at 3 months, further improvements at 6 months were observed for median (IQR) symptom scores including the TSS (8.0 [2.0-10.0] vs 18 [14.0-21.0]; *P* < .001), the GCSI (1.2 [0.4-2.5] vs at baseline: 3.3 [2.8-4.1]; *P* < .001), and the PAGI-SYM (1.3 [0.3-1.8] vs 2.9 [2.1-3.8]; *P* < .001) and for HLOS (0 [ 0-2.0] vs 4.1 [0-10.1]; *P* = .05). Significant improvements in symptom scores were observed in 6 months compared with the 3-month values (Table 3). These findings were mostly consistent in parametric data analysis using repeated-measures ANOVA followed by Bonferroni correction (eTable 5 in Supplement 2).

**Table 3.** Outcome Measures in the PP + GES-OFF Group at 3 Months and 6 Months After Surgery^a^

Factor	Median (IQR)			*P* value^b^		
	Baseline	3 mo	6 mo	Baseline vs 3 mo (PP effect)	Baseline vs 6 mo (PP + GES effect)	3 mo vs 6 mo (GES effect)
Total symptom score^c^						
Total	18.0 (14.0-21.0)	12.0 (6.0-18.0)	8.0 (2.0-10.0)	.006^d^	<.001^d^	.001
Vomiting	3.0 (3.0-4.0)	3.0 (1.0-4.0)	1.0 (0-2.0)	.21	.001^d^	.02
Nausea	3.5 (3.0-4.0)	2.5 (1.0-3.0)	1.0 (0-2.0)	.07	.001^d^	.04
Early satiety	3.0 (3.0-4.0)	2.0 (1.0-3.0)	1.0 (0-2.0)	.19	.005^d^	.006
Bloating	3.0 (2.0-4.0)	2.0 (0-3.0)	1.0 (0-2.0)	.12	.001^d^	.06
Postprandial fullness	3.0 (2.0-4.0)	2.0 (1.0-3.0)	1.0 (0-2.0)	.04	.007^d^	.29
Epigastric pain	3.0 (2.0-4.0)	2.0 (0-3.0)	0 (0-1.0)	.09	.005^d^	.15
Gastroparesis Cardinal Symptom Index^e^						
Total	3.3 (2.8-4.1)	2.3 (1.2-3.1)	1.2 (0.4-2.5)	.002^d^	<.001^d^	.001
Nausea or vomiting	3.8 (3.3-4.7)	2.7 (1.7-3.3)	1.3 (0.7-2.3)	.007^d^	<.001^d^	.004
Postprandial fullness	3.6 (2.3-4.3)	1.9 (1.3-2.8)	1.5 (0.5-2.8)	.02	.008	.11
Bloating	3.8 (1.5-5.0)	2.3 (1.0-3.0)	1.0 (0-2.0)	.12	.02	.02
PAGI-SYM^f^	2.9 (2.1-3.8)	1.8 (0.9-2.7)	1.3 (0.3-1.8)	<.001^d^	<.001^d^	<.001
Hemoglobin A_1c_ in DMGP	8.8 (7.3-10.7)	9.5 (6.9-11.2)	7.8 (6.4-8.4)	1.00	.19	>.99
Hospitalization, d	4.1 (0-10.1)	0 (0-4.0)	0 (0-2.0)	.18	.05	>.99
Presence of hospitalizations, No. (%)	11 (69)	5 (29)	4 (29)	.08^d^	.04^d^	>.99
Gastric emptying retention, %						
At 2 h	69.0 (60.0-80.0)	38.0 (28.0-48.0)	NA	.001^d^	NA	NA
At 4 h	38.5 (27.0-57.0)	10.0 (7.0-25.0)	NA	.002^d^	NA	NA

Abbreviations: DMGP, diabetic gastroparesis; GES, gastric electrical stimulation; NA, not applicable; PAGI-SYM, Patient Assessment of Upper Gastrointestinal Disorders Symptom Severity Index; PP, pyloroplasty.

^a^ In the GES-OFF group, the device remained inactive until 3 months after surgery. Nonparametric Friedman test analysis was followed by the Wilcoxon signed rank test after adjusting for Bonferroni correction for multiple comparisons.

^b^ Calculated using the Wilcoxon signed rank test, except for hospitalization, using McNemar tests.

^c^ Scores range from 0 to 24, with higher scores indicating more severe symptoms.

^d^ Result remained significant at 1% alpha even after correcting for multiplicity due to multiple outcomes.

^e^ Scores range from 0 to 5, with higher scores indicating more severe symptoms.

^f^ Scores range from 0 to 5, with higher scores indicating more severe symptoms.

### Effects of PP + GES-ON vs PP + GES-OFF Groups on Outcomes at 6-Month Visit

Median changes in symptom scores measured at the 6-month visit vs baseline in the PP + GES-OFF group were not different than the results achieved by those in the PP + GES-ON group whose system was turned on continuously for 6 months (eTable 6 in Supplement 2). These findings were mostly consistent in parametric data analysis using repeated-measures ANOVA followed by Bonferroni correction (eTable 7 in Supplement 2).

### Complications and Serious Adverse Events

No immediate postoperative problems or major adverse events were observed following the combined GES and PP surgeries. There were 2 deaths unrelated to treatment. One patient developed diabetes ketoacidosis due to noncompliance approximately 6 weeks after the surgery, and the other death was due to a narcotic overdose a few weeks after the operation.

## Discussion

The current study is the first randomized clinical trial, to our knowledge, that has assessed the effectiveness of combining PP and GES in refractory gastroparesis. The study demonstrated a significant improvement in gastroparesis symptoms in the PP + GES-ON group compared with PP alone (GES-OFF) after 3 months, which was maintained at the 6-month follow-up, accompanied by a reduction in HLOS and HbA_1c_ (in DMGP). PP alone without GES (GES-OFF) resulted in a significant reduction in gastroparesis symptom scores, but with activation of the GES after 3 months, there was a further significant improvement in symptoms by 6 months, achieving outcomes comparable with the group whose GES had been activated for the whole duration of the trial. At 3 months, both groups demonstrated greater reduction in the TSS, the GCSI, and the PAGI-SYM, even to a greater extent than the specified minimal clinically important differences in our study.

Effective medical treatments for refractory gastroparesis remain limited, with metoclopramide being the only US Food and Drug Administration-approved drug, although adverse effects limit its use.^17,18^ Therefore, endoscopic and surgical interventions have gained attention. While PP aims to accelerate GE, potentially improving symptoms such as fullness and early satiety, GES would alleviate nausea and vomiting through central nervous system effects and vagal modulation, reduce gastric hypersensitivity, and improve fundic accommodation.^8^

Improvement of gastroparesis symptoms with PP supports the concept of pyloric dysfunction as a part of gastroparesis. Multiple studies have demonstrated modest to substantial symptom improvement and GE after G-POEM or PP, although some patients required additional interventions.^19,20^ A G-POEM meta-analysis involving 482 patients with gastroparesis found a modest clinical success of 61% based on pooled rates (95% CI).^21^ Besides G-POEM, PP as a stand-alone surgical procedure has demonstrated improvements in objective measures of GE time and a low risk of complications.^22,23^ Shada et al^24^ studied 177 patients who underwent laparoscopic Heineke-Mikulicz PP. GE was improved or normalized in nearly 90% of patients. Surgery also improved symptoms of nausea, vomiting, bloating, and abdominal pain. However, up to 11% of those with PP subsequently required additional interventions, including subtotal gastrectomy, placement of gastrojejunostomy tubes, or implantation of a gastric stimulation system. In another study of 26 patients with gastroparesis who underwent laparoscopic PP and 2 with laparoscopic-assisted, flexible transoral endoscopic circular-stapled PP, prokinetic use was significantly reduced, GE significantly improved, and the symptom severity score for nausea, vomiting, bloating, abdominal pain, and gastroesophageal reflux disease symptoms improved. Significant improvement persisted at 3 months for nausea, vomiting, bloating, abdominal pain, and reflux symptoms.^25^ In a different study on PP, symptom improvement was reported by 82%, and GE was significantly improved. Some patients required further procedures later: distal gastrectomy (n = 2), duodenojejunostomy (n = 2), and gastric stimulator placement (n = 1).^22^ It is well established that PP and pyloromyotomy share similar limitations with G-POEM in that they do not address the overall gastric dysmotility in gastroparesis.

GES typically does not alter GE but improves symptoms using neuromodulation. In the ON group, similar GE improvements to the OFF group highlight that symptom differences likely stem from central or visceral modulation, not from emptying. Positron emission tomography scans of the brain suggest that GES affects the central nervous system by modulating the chemoreceptor trigger zone and activating vagal afferent pathways.^26^ Additionally, GES increases vagal efferent autonomic activity and reduces gastric sensitivity to volume distention, resulting in improved fundic relaxation and accommodation.^27^ GES may enhance the growth of interstitial cells of Cajal, controlling the regularity of the electrical signal in the stomach.^28^ Other researchers have reported that after GES, there was an increase in interstitial cells of Cajal counts over time, consistent with findings of improvement in clinical symptoms and GE.^29^

Studies that supported the approval of GES have concentrated on device implantation without pyloric intervention. One study showed that during a 12-month follow-up, there was a 36% reduction in the TSS, a 75% reduction in hospitalization days, and a 68% decrease in the frequency of weekly vomiting episodes in patients who were previously documented with diabetic medication-refractory gastroparesis.^30^ In another study of GES for IDGP, there was an 87% reduction in the frequency of weekly vomiting with a GES-ON group at 1 year, a 100% reduction in hospitalization, and a 40% reduction in the TSS.^31^ Additionally, 3 long-term follow-up studies, extending up to 10 years, confirmed that GES remains effective for patients with gastroparesis with refractory nausea and vomiting, showing efficacy in 50% of cases.^10,32,33^ In a study of a total of 157 patients who had undergone GES implantation, all 9 gastroparesis symptom components of the GCSI, as well as the total GCSI score, were significantly decreased at 1 year postoperatively with sustained effects at the 5-year follow-up.^34^

Studies evaluating the combination of GES and PP are limited. One study assessed the effectiveness of GES alone, PP alone, and GES with PP in improving nausea and vomiting and found that the GCSI nausea or vomiting subscale was significantly improved with the combination of GES and PP, whereas PP alone was not effective.^11^ Additionally, a previous study by our team involving 24 patients receiving GES with PP, with follow-up extending up to 38 months, reported a 71.0% improvement in total symptom scores and a 48.7% reduction in radiolabeled meal retention at 4 hours.^12^ Another study found that symptom improvement in refractory gastroparesis appears to improve after placement of a GES device with or without the addition of PP.^35^ A recent step-up study of patients with gastroparesis, in which GES was offered to patients with a suboptimal response to PP, found a significant decrease in the GCSI score and the retention of a radiolabeled meal in 4-hour GE with PP alone. An adjunct GES was required in 28.8% of cases. Those with suboptimal responses to PP were younger, with a higher baseline GCSI. After GES, the GCSI and GE improved and were comparable with those who only required PP.^36^ Compared with previous studies,^11,35^ although the trend of improvements remained consistent across groups, the effect size for primary outcomes was relatively larger in our study. The benefits of the treatments seem to be additive rather than synergistic in our study. We observed that turning on the GES device further improved outcomes, including the symptom scores. Moreover, the percentage improvements in the outcome measures were observed to a greater extent with the GES device on compared with PP alone, indicating more additive effects than synergistic effects. This was also confirmed in another study that demonstrated improved outcomes following GES in failed PP.^35^

PP with GES did not cause a major serious adverse event associated with the procedure in our study. A retrospective study showed surgical-site infection in 7 patients (26%) of the combination therapy vs in 4 patients (6%) of the GES-only treatment.^37^ In another study of 157 patients with GES implantation, devices were explanted in 5 patients, 12 patients required battery exchanges, and 7 patients required reoperation due to displaced or eroded device leads.^34^

### Strengths and Limitations

The study’s strengths included a double-blind randomized design, a GES-OFF control group, and sufficient power to detect symptom improvements, with a lower-than-expected dropout rate. Effect sizes for key outcomes were robust.

The study also has limitations. These include a small sample of patients with IDGP, limiting subgroup analysis; a lack of data on antiemetic use; and the need for longer follow-up to assess long-term effects. Variations in surgical techniques, GES programming, dietary protocols, and surgeon experience may affect the generalizability of the results.

## Conclusions

The findings of this randomized clinical trial suggest that combined PP and GES is safe and effective in refractory gastroparesis. GES increases symptomatic relief, and while immediate activation leads to earlier maximal benefit, delayed activation of GES after PP can achieve equivalent symptomatic control. The integration of diagnostic techniques, such as recording stomach electrical activity and the assessment of pyloric diameter and distensibility, may enhance patient profiling and optimize decision-making for pyloric treatments and/or GES.

## Notes

Supplement 1.
Trial Protocol

Supplement 2.
eMethods
**eTable 1.** Median Changes in Outcome Measures at 3 Months in PP+GES-ON and PP+GES-OFF Groups Using the Wilcoxon Rank Sum Test **eTable 2.** Unadjusted and Adjusted Differences in Improvement in Primary Outcomes at 3 Months Between the PP+GES-ON and PP+GES-OFF Groups Using Median Regressions **eTable 3.** Comparisons of Mean Changes in Outcome Measures From Baseline at 3 Months Between the PP+GES-ON and PP+GES-OFF Groups **eTable 4.** The Trend in the Outcome Measures in the PP+GES-ON Group **eTable 5.** Effects of PP Alone and PP+GES on the Outcome Measures in the PP+GES-OFF Group **eTable 6.** Median Changes in Outcome Measures at 6 Months in PP+GES-ON and PP+GES-OFF Groups **eTable 7.** Comparison of Mean Changes in Outcome Measures at 6 Months Between PP+GES-ON and PP+GES-OFF Groups **eFigure.** Median GCSI Subscale Scores Representing PP+ GES-ON vs PP+GES-OFF Groups
eReferences

Supplement 3.
Data Sharing Statement

## References

[1] Parkman HP, Hasler WL, Fisher RS; American Gastroenterological Association. American Gastroenterological Association technical review on the diagnosis and treatment of gastroparesis. Gastroenterology. 2004;127(5):1592-1622. doi:10.1053/j.gastro.2004.09.055 15521026

[2] Ye Y, Yin Y, Huh SY, Almansa C, Bennett D, Camilleri M. Epidemiology, etiology, and treatment of gastroparesis: real-world evidence from a large US national claims database. Gastroenterology. 2022;162(1):109-121.e5. doi:10.1053/j.gastro.2021.09.064 34624355

[3] Moshiree B, Douglas Y. Clinical presentations of gastroparesis. In: McCallum RW, Parkman HP, eds. Gastroparesis. Academic Press; 2021:19-33. doi:10.1016/B978-0-12-818586-5.00003-X

[4] Rayner CK, Jones KL, Horowitz M. Diabetic gastroparesis. In: McCallum RW, Parkman HP, eds. Gastroparesis. Academic Press; 2021:237-253. doi:10.1016/B978-0-12-818586-5.00018-1

[5] Gonzalez Z, McCallum RW. Idiopathic gastroparesis. In: McCallum RW, Parkman HP, eds. Gastroparesis. Academic Press; 2021:265-281. doi:10.1016/B978-0-12-818586-5.00020-X

[6] Sarosiek I, McCallum R. Gastric electrical stimulation for gastroparesis. In: McCallum RW, Parkman HP, eds. Gastroparesis. Academic Press; 2021:413-429. doi:10.1016/B978-0-12-818586-5.00030-2

[7] Shanker A, Bashashati M, Rezaie A. Gastric electrical stimulation for treatment of refractory gastroparesis: the current approach to management. Curr Gastroenterol Rep. 2021;23(2):2. doi:10.1007/s11894-020-00803-0 33483775 PMC7822763

[8] Davis BR, McCallum RW. Surgical management of gastroparesis. In: McCallum RW, Parkman HP, eds. Gastroparesis. Academic Press; 2021:431-439. doi:10.1016/B978-0-12-818586-5.00031-4

[9] Abidi N, Starkebaum WL, Abell TL. An energy algorithm improves symptoms in some patients with gastroparesis and treated with gastric electrical stimulation. Neurogastroenterol Motil. 2006;18(4):334-338. doi:10.1111/j.1365-2982.2006.00765.x 16553589

[10] McCallum RW, Lin Z, Forster J, Roeser K, Hou Q, Sarosiek I. Gastric electrical stimulation improves outcomes of patients with gastroparesis for up to 10 years. Clin Gastroenterol Hepatol. 2011;9(4):314-319.e1. doi:10.1016/j.cgh.2010.12.013 21185396

[11] Zoll B, Jehangir A, Edwards MA, . Surgical treatment for refractory gastroparesis: stimulator, pyloric surgery, or both? J Gastrointest Surg. 2020;24(10):2204-2211. doi:10.1007/s11605-019-04391-x 31512100

[12] Davis BR, Sarosiek I, Bashashati M, Alvarado B, McCallum RW. The long-term efficacy and safety of pyloroplasty combined with gastric electrical stimulation therapy in gastroparesis. J Gastrointest Surg. 2017;21(2):222-227. doi:10.1007/s11605-016-3327-4 27896652

[13] Dwivedi AK, Shukla R. Evidence-based statistical analysis and methods in biomedical research (SAMBR) checklists according to design features. Cancer Rep (Hoboken). 2020;3(4):e1211. doi:10.1002/cnr2.1211 32794640 PMC7941456

[14] Abell TL, Camilleri M, Donohoe K, ; American Neurogastroenterology and Motility Society and the Society of Nuclear Medicine. Consensus recommendations for gastric emptying scintigraphy: a joint report of the American Neurogastroenterology and Motility Society and the Society of Nuclear Medicine. J Nucl Med Technol. 2008;36(1):44-54. doi:10.2967/jnmt.107.048116 18287197

[15] Revicki D, Parkman H. Evaluating response in gastroparesis: patient reported outcome measures and survey instruments. In: McCallum RW, Parkman HP, eds. Gastroparesis. Academic Press; 2021:451-460. doi:10.1016/B978-0-12-818586-5.00033-8

[16] Dwivedi AK. How to write statistical analysis section in medical research. J Investig Med. 2022;70(8):1759-1770. doi:10.1136/jim-2022-002479 35710142 PMC9726973

[17] Sarosiek I, Bashashati M, McCallum RW. Safety of treatment for gastroparesis. Expert Opin Drug Saf. 2016;15(7):937-945. doi:10.1517/14740338.2016.1173204 27031006

[18] Kalas MA, Trivedi B, Kalas M, Chavez LO, McCallum RW. Metoclopramide in gastroparesis: its mechanism of action and safety profile. *Gastrointest Disord*. 2023;5(3):317-328. doi:10.3390/gidisord5030026

[19] Martinek J, Hustak R, Mares J, . Endoscopic pyloromyotomy for the treatment of severe and refractory gastroparesis: a pilot, randomised, sham-controlled trial. Gut. 2022;71(11):2170-2178. doi:10.1136/gutjnl-2022-326904 35470243 PMC9554080

[20] Vosoughi K, Ichkhanian Y, Benias P, . Gastric per-oral endoscopic myotomy (G-POEM) for refractory gastroparesis: results from an international prospective trial. Gut. 2022;71(1):25-33. doi:10.1136/gutjnl-2020-322756 33741641

[21] Kamal F, Khan MA, Lee-Smith W, . Systematic review with meta-analysis: one-year outcomes of gastric peroral endoscopic myotomy for refractory gastroparesis. Aliment Pharmacol Ther. 2022;55(2):168-177. doi:10.1111/apt.16725 34854102

[22] Toro JP, Lytle NW, Patel AD, . Efficacy of laparoscopic pyloroplasty for the treatment of gastroparesis. J Am Coll Surg. 2014;218(4):652-660. doi:10.1016/j.jamcollsurg.2013.12.024 24529808

[23] Petrov RV, Bakhos CT, Abbas AE, Malik Z, Parkman HP. Endoscopic and surgical treatments for gastroparesis: what to do and whom to treat? Gastroenterol Clin North Am. 2020;49(3):539-556. doi:10.1016/j.gtc.2020.04.008 32718569 PMC7391056

[24] Shada AL, Dunst CM, Pescarus R, . Laparoscopic pyloroplasty is a safe and effective first-line surgical therapy for refractory gastroparesis. Surg Endosc. 2016;30(4):1326-1332. doi:10.1007/s00464-015-4385-5 26293794

[25] Hibbard ML, Dunst CM, Swanström LL. Laparoscopic and endoscopic pyloroplasty for gastroparesis results in sustained symptom improvement. J Gastrointest Surg. 2011;15(9):1513-1519. doi:10.1007/s11605-011-1607-6 21720926

[26] McCallum RW, Dusing RW, Sarosiek I, Cocjin J, Forster J, Lin Z. Mechanisms of symptomatic improvement after gastric electrical stimulation in gastroparetic patients. *Neurogastroenterol Motil*. 2010;22(2):161-e51. doi:10.1111/j.1365-2982.2009.01389.x 19719511

[27] Gourcerol G, Ouelaa W, Huet E, Leroi AM, Ducrotte P. Gastric electrical stimulation increases the discomfort threshold to gastric distension. Eur J Gastroenterol Hepatol. 2013;25(2):213-217. doi:10.1097/MEG.0b013e32835a7f04 23075698

[28] Pontikos A, Jayakumar P, Rios Perez C, . Gastric electrical stimulation has an effect on gastric interstitial cells of Cajal (ICC) that is associated with mast cells. Cureus. 2020;12(11):e11458. doi:10.7759/cureus.11458 33329956 PMC7733771

[29] Sarosiek I, Bashashati M, Torelli R, Padilla O, Davis B, McCallum R. S1907: Effect of chronic gastric electrical stimulation on regeneration of gastric interstitial cells of Cajal in gastroparesis patients. Am J Gastroenterol. 2023;118(10S):S1415-S1416. doi:10.14309/01.ajg.0000957268.97929.e8

[30] McCallum RW, Snape W, Brody F, Wo J, Parkman HP, Nowak T. Gastric electrical stimulation with Enterra therapy improves symptoms from diabetic gastroparesis in a prospective study. Clin Gastroenterol Hepatol. 2010;8(11):947-954. doi:10.1016/j.cgh.2010.05.020 20538073

[31] McCallum RW, Sarosiek I, Parkman HP, . Gastric electrical stimulation with Enterra therapy improves symptoms of idiopathic gastroparesis. Neurogastroenterol Motil. 2013;25(10):815-e636. doi:10.1111/nmo.12185 23895180 PMC4274014

[32] Hedjoudje A, Huet E, Leroi AM, Desprez C, Melchior C, Gourcerol G. Efficacy of gastric electrical stimulation in intractable nausea and vomiting at 10 years: a retrospective analysis of prospectively collected data. Neurogastroenterol Motil. 2020;32(11):e13949. doi:10.1111/nmo.13949 33107679

[33] van der Voort IR, Becker JC, Dietl KH, Konturek JW, Domschke W, Pohle T. Gastric electrical stimulation results in improved metabolic control in diabetic patients suffering from gastroparesis. Exp Clin Endocrinol Diabetes. 2005;113(1):38-42. doi:10.1055/s-2004-830525 15662594

[34] Cassidy DJ, Gerull W, Zike VM, Awad MM. Clinical outcomes of a large, prospective series of gastric electrical stimulation patients using a multidisciplinary protocol. J Am Coll Surg. 2024;239(4):341-346. doi:10.1097/XCS.0000000000001105 38682813 PMC11392156

[35] Bauzon J, Wang MY, Barber AE. Addition of pyloroplasty may improve glycemic control and refractory early satiety in gastroparesis at rates similar to gastric neurostimulation alone: a retrospective analysis. Scand J Gastroenterol. 2024;59(9):1035-1038. doi:10.1080/00365521.2024.2386038 39105565

[36] Eriksson SE, Gardner M, Sarici IS, . Efficacy of gastric stimulator as an adjunct to pyloroplasty for gastroparesis: characterizing patients suitable for single procedure vs dual procedure approach. J Gastrointest Surg. 2024;28(11):1769-1776. doi:10.1016/j.gassur.2024.08.007 39127405

[37] Paturu T, Englander K, Ganam S, Velanovich V, Sujka J. Infection rate in patients after Enterra device placement with concurrent pyloroplasty. J Gastrointest Surg. 2025;29(6):102056. doi:10.1016/j.gassur.2025.102056 40210084

