# Supplementary material for: Combined Gastric Electrical Stimulation and Pyloroplasty in Gastroparesis: A Randomized Clinical Trial
Source: JAMA Netw Open. 2025 Dec 9;8(12):e2546332. doi: 10.1001/jamanetworkopen.2025.46332 (PMC12690432; doi:10.1001/jamanetworkopen.2025.46332)
Supplement: Supplement 1. — Trial Protocol [file jamanetwopen-e2546332-s001.pdf]

Title of Project: Combined gastric electrical stimulation (GES) and pyloroplasty for the treatment of gastroparesis: Can pyloroplasty be effective without GES? – A double-blind trial.

Version 11/20/2018

Principal Investigator: Irene Sarosiek, M.D. TTUHSC Department of Internal Medicine

Co-Investigator: Richard W. McCallum, M.D. TTUHSC Department of Internal Medicine

Co-Investigator: Brian R. Davis, M.D. TTUHSC Department of Surgery

Co-Investigator: Alireza Torabi, M.D., Ph.D. TTUIHSC Department of Pathology

Co-Investigator: Jesus R. Diaz, MD TTUHSC Department of Radiology/ Nuc. Medicine

Co-Investigator: Mohammad Bashashati, M.D.TTUHSC Department of Internal Medicine

Abstract:

Gastroparesis (GP), defined as delayed gastric emptying without any mechanical obstruction affects up to 10 million individuals in the United States. Improvement of symptoms is achieved in up to 50-60% of drug refractory patients treated with gastric electrical stimulation (GES), but this therapy has minimal or no effect on the acceleration of gastric emptying (GE). To address this therapeutic deficiency, we have added surgical pyloroplasty (PP) as a supplementary procedure to accelerate GE in drug refractory gastroparetics undergoing implantation of GES. Based on our experience, there was more than 70% improvement in total GP symptom scores (TSS) in the follow-up evaluation and GE was normalized in 60 % of GP patients who received combined GES and pyloroplasty, suggesting that the combination of PP and GES significantly accelerate gastric emptying and improve GP symptoms exceeding the results previously achieved by GES alone. Now, the question is whether pyloroplasty alone could be sufficient for the achievement of both subjective and objective goals of improvement in drug refractory gastroparesis. Therefore, in the current proposal, we plan to compare TSS outcome, quality of life and GE in drug refractory GP patients who will receive both GES implantation and PP during the surgery and then are evaluated during a GES–off or –On period based on a randomized double blind study design.

As a secondary goal, we will also examine whether the baseline pyloric and antral interstitial cells of Cajal (ICC) counts as well as the presence of pyloric fibrosis could predict clinical response to PP.

Research Plan: A) Specific Aims

1) To assess the effects of gastric electrical stimulation (GES) and pyloroplasty on total GP symptoms scores (TSS) and quality of life in drug refractory gastroparetic patients during GES -ON and GES -OFF periods.

2) To assess the effects of gastric electrical stimulation (GES) and pyloroplasty on gastric emptying of a radiolabeled marker in drug refractory gastroparetic patients during GES-ON and GES -OFF periods.

3) To understand whether pyloroplasty alone is effective for achieving both subjective and objective goals of improvement in drug refractory gastroparesis.

4) To understand whether the status of antral and pyloric ICC counts and/or the presence of pyloric fibrosis predicts the clinical outcome.

B) Background/significance:

Gastroparesis (GP), affecting up to 10 million individuals in the United States, is a relatively common gastrointestinal (GI) motility disorder and is defined as delayed gastric emptying without any mechanical obstruction. Gastroparesis presents with upper GI symptoms such as nausea, vomiting, bloating, postprandial fullness, early satiety and abdominal pain. The etiology of gastroparesis is not well recognized and the majority of cases are idiopathic (ID-GP), while many others are diabetic (DM-GP). Patients with gastroparesis suffer from nutritional deficiencies and metabolic consequences as well as impaired social activities and quality of life. Treatment of gastroparesis is based on alleviating symptoms, correcting nutritional abnormalities and targeting the underlying causes, although it is usually challenging and often disappointing [1]. Therefore, studying the treatment options of this debilitating disorder are among the priorities in the field of GI motility disorders.

Clinically gastroparesis is categorized as mild, moderate and severe. Mild gastroparesis presents with occasional symptoms which do not have a significant impact on work and family functioning. The treatment in this group is based on diet modification, antiemetics and glucose control. Moderate gastroparesis presents with daily but not continuous symptoms and occasional hospitalization, interfering with work and family functioning. Diet modifications, prokinetics, one or more antiemetics and glucose control as well as addressing pain and psychological aspects are

recommended in these patients. In severe Gastroparesis daily continuous symptoms are present resulting in multiple hospitalizations and inability to work and function. Severe gastroparesis is treated with combining prokinetics, multiple antiemetics and nutrition enteral support. Up to 30% of gastroparetic patients fail current medical therapy and need surgery including gastric electrical stimulation (GES) implantation and/or pyloroplasty as the next step[1, 2].

GES is indicated for the treatment of chronic, intractable nausea and vomiting secondary to diabetic or idiopathic gastroparesis since 2000 under a Humanitarian Device Exemption (HDE) and involves implantation of a pulse generator in the abdominal wall and 2 electrodes into the muscularis propria of the stomach. Based on our experience as well as other national centers, the improvement of symptoms is variable with the maximal response up to 50-60%, but this therapy has minimal or no effect on the acceleration of gastric emptying (GE).[3]

Pyloric dysfunction is recognized as a significant component of gastroparesis. Pylorospasm has been hypothesized to be present in both diabetic GP and ID-GP based on pyloric dysfunction identified by pyloric motility findings. In addition, the lack of acceleration of the delayed GE by GES raises the question as how much better the outcome would be if gastric emptying could be accelerated. This is the rationale for the addition of a surgical pyloroplasty performed at the time when GES is implanted. Surgical papers have suggested that pyloroplasty alone could have a role in patients with GP. Our recent study on pyloroplasty combined with GES showed that GE was normalized in 60% of patients with GP. Patients who received only GES therapy decreased their TSS severity score by <50%, while those patients receiving pyloroplasty and GES had improvement in the severity of gastroparetic symptoms by>70% 3, 4.

Despite these findings, the question could be asked whether pyloroplasty alone without GES could generate similar results to GES plus pyloroplasty. To answer this question, a double-blind and randomized study should be performed on GP patients who receive combined GES implantation and pyloroplasty. Severity and frequency of gastroparesis symptoms, quality of life, hospitalization, changes in antiemetic/prokinetic and analgesic medications and glucose control parameters in diabetes (HbA1c) will be assessed during: (a) a baseline pre-op period, (b) on a day of surgery, (c) 3 months GES-ON and -OFF periods and (d) last follow up visit.
Gastric emptying with a radiolabeled meal will performed as a standard of care test, the way it is

conducted now on all patients receiving GES.

Additionally, as reported, approximately 40% of GP patients who are refractory to medical therapy and requiring GES therapy have a depletion of antral ICC. Moreover, our recent research has revealed that more than 70% of these patients show depletion of pyloric ICC. Therefore, we would like to assess whether pyloric and antral ICC counts could predict response to pyloroplasty during GES-ON and -OFF periods [5].

This concept of our study requires patients to sign, the specific IRB-approved consent form for E14018 study in order to provide a tissue sample as it is described under that protocol.

C) Preliminary data

C1) GES-ON plus Pyloroplasty:

A pilot study [abstract submitted to DDW 2016] was designed to assess the long term efficacy (follow-up visits: 3 to 38 months) and safety of combined GES implantation and PP in GP patients who were referred to our clinic from September 2012 to June 2015. Twenty-seven [23 females; mean age 43 years old (23–63); mean weight 148 lbs (86–245)] drug-refractory GP patients who underwent surgical implantation of the GES together with the Heineke-Mikulicz PP during the study period were included. There were 17 diabetics (DM) and 10 idiopathics (IP). There was ~71% improvement in TSS in the follow-up evaluation (Table 1). After surgery, the mean retention of the radiolabeled meal decreased by 29.6% and 48.7% at 2 and 4-hrs, respectively and GE was normalized in 60 % of GP patients. There were no post-surgical complications or technical problems related to combining PP with GES.

Table 1) The severity of upper gastrointestinal symptoms in gastroparetic patients at baseline and after surgery (gastric electrical stimulator implantation plus pyloroplasty).

	Nausea	Vomiting	Early Satiety	Bloating	Post-Prandial Fullness	Epigastric Pain	Epigastric Burning
Pre-Op*	3.6 (0.4)	3.2 (1.2)	3.2 (0.7)	2.5 (1.3)	2.8 (0.9)	2.7 (1.4)	2.0 (1.8)
Post-Op*	1.1 (1.1)***	0.6 (1.0)***	0.9 (1.0)***	0.9 (1.2)**	1.0 (1.1)***	1.0 (1.5)***	0.9 (1.5) †

* data represents mean (standard deviation),**P<0.01, ***P<0.001, †n's. not significant

C2) GES-ON without pyloroplasty:

Gastroparesis patients (n=221; 142 diabetic, 48 idiopathic, and 31 postsurgical) treated with Enterra for 1-11 years were retrospectively assessed; 188 had follow-up visits and data were collected for at least 1 year. TSS, hospitalization days, and use of medications were significantly reduced among all patients. More patients with diabetic (58%) and postsurgical gastroparesis (53%) had a greater than 50% reduction in TSS than those with idiopathic disease (48%). Weight significantly increased among all groups, and 89% of J-tubes could be removed. At end of the follow-up period, all etiological groups had similar, abnormal delays in mean gastric retention. Thirteen patients (7%) had their devices removed because of infection at the pulse generator site. There was ~48% improvement in TSS in the follow-up evaluation, while mean follow-up GE was not significantly different compared to the baseline indicating that **GES alone** does not significantly accelerate gastric emptying (Table 2)[6].

Table 2) Comparison of Individual Symptom Scores (Mean ± SD) Between Baseline and Follow-Up (gastric electrical stimulator implantation only)

	Nausea	Vomiting	Early Satiety	Bloating	Post-Prandial Fullness	Epigastric Pain	Epigastric Burning
Pre-Op19.6	3.5 (1.6)	3 (1.2)	2.9 (1.1)	2.8 (1.2)	2.8 (1.1)	2.5 (1.3)	2.1 (1.4)
Post-Op	1.6 (1.3) ***	1.4 (1.3) ***	1.5 (1.3) ***	1.4 (1.3) ***	1.4 (1.2) ***	1.3 (1.3) ***	0.8 (1.1) ***

* data represents mean (standard deviation), ***P<0.001

D) Research Plan

D1) Patients: the criteria for patient's selection will be based on a goal to achieve an adequate number of patients from 2 major etiological subgroups of GP (diabetics and idiopathic) among all potential GES candidates. Adult patients (18-70 years old) will be approached regarding this research trial.

Inclusion criteria:

Documented diagnosis of GP for > 1 year and refractoriness to anti-emetics and prokinetics;
more than 7 emetic episodes per week; and delayed GE (gastric retention greater than 60% at 2 h and/or greater than 10% at 4 h) based on a 4-h standardized radionuclide solid meal test.

Exclusion Criteria:

Organic or pseudo-obstruction, primary eating or swallowing disorders, positive pregnancy test result, psychogenic vomiting, peritoneal dialysis, drug dependent, morbid obesity, active malignancy and whoever received PP or GES in the past.

D2) Sample Size: As this study is designed to conclude that pyloroplasty is not inferior to GES plus pyloroplasty, the sample size for the non-inferiority trial by including % normalization of the GE as the binary outcome is calculated based on: "Blackwelder WC."Proving the Null Hypothesis" in Clinical Trials. Control. Clin. Trials 1982; 3:345-353." GE was normalized in 60 % of GP patients with GES-ON

plus Pyloroplasty based on our preliminary data. On the other hand, based on our patients' database, GES alone normalizes GE in around 18% of GP patients, predominantly in idiopathic GP. By defining non- inferiority limit equal to (60 - 18= 42%), and percentage of success in the GES-ON plus pyloroplasty group, 17 patients will be included in each arm of this study. Therefore, this study is designed to include overall 34 drug-refractory GP patients who will undergo surgery for GES implantation plus pyloroplasty. Based on our records, we have at least 2 surgeries per month; therefore, we predict that it takes approximately 1.5 years to recruit all patients.

D3) Study Design: After receiving IRB approval, the patients will be screened and the project will be discussed with the potential candidates for GES implantation plus pyloroplasty. Based on the inclusion/exclusion criteria, the baseline gastric emptying test (GET) based on the 4-h standardized radionuclide solid meal protocol will be performed and 34 patients will be included in the study. Our effort would be to include similar numbers of idiopathic and diabetic patients in each intervention group based on a block randomization method. The patients will be followed up for 1 month, while their PAGI-SYM, PAGI-QOL and TSS severity/frequency will be recorded on a Standardized Symptoms Interview Form, which assesses the symptoms of gastroparesis occurring during the last 2 weeks before the interview for severity of vomiting, nausea, early satiety, bloating, postprandial fullness, and epigastric pain. The severity of each symptom will be graded by the patients as 0=absent, 1=mild (not influencing usual activities), 2=moderate (diverting from, but not urging modifications, of usual activities), 3=severe (influencing usual activities, severely enough to urge modifications), and 4=extremely severe (requiring bedrest). The sum of the severity ratings of the six symptom subscores comprises the overall total symptom score (TSS) in severity.

PAGI- SYM and TSS questionnaires will be filled every 2 weeks during the study period. GES implantation together with the Heineke-Mikulicz PP will be performed laparoscopically (<10% of cases may require open approach). Only one surgeon using the same technique at all times will perform the surgeries. In GES-ON group, GES will be turned on immediately after the operation. On the other hand, in GES-OFF group, the GES will remain OFF for the first 3 months, while symptoms will be recorded again in both groups. After 3 months, follow up GET will be performed and in GES-OFF group, the device will be turned ON. Both groups will be followed for 3 more months clinically, to record any possible changes in their symptoms. Moreover, 36-Item Short Form Health Survey (SF-36), Beck depression inventory (BDI), State-trait anxiety inventory (STAI) and the patient health questionnaire (PHQ-15) will be completed at baseline followed by 3 and 6 months after the surgery. The amount of anti-emetics and prokinetics, days of hospitalization and ED visits and any possible complication will be recorded during the study period. Glucose control in diabetic patients will be monitored by HbA1c. Pain will also be monitored regarding narcotic need. During the follow-up patients will be instructed to remain on a mechanical soft diet of smaller meals, low fat, and low fiber. Evaluators of clinical outcome, the radiologist and the biostatistics consultant as well as the patient will remain blind to the GES-ON or –OFF status before the results get finalized. The individual programming the device will have no clinical rule to avoid any bias.

One of our Investigators, Dr. Mohammad Bashashati is assigned to serve as an un-blind person , who is going to generate a master list of participants by dividing them into two groups based on their etiologies (diabetic or idiopathic). All efforts would be to include similar numbers of idiopathic and diabetic patients in each intervention group based on a block randomization method, allowing for GES to be turned ON at the surgery, or it will stay OFF for the 3-month-long blind portion of the study.

The following flow-diagram summarizes the steps of this study:

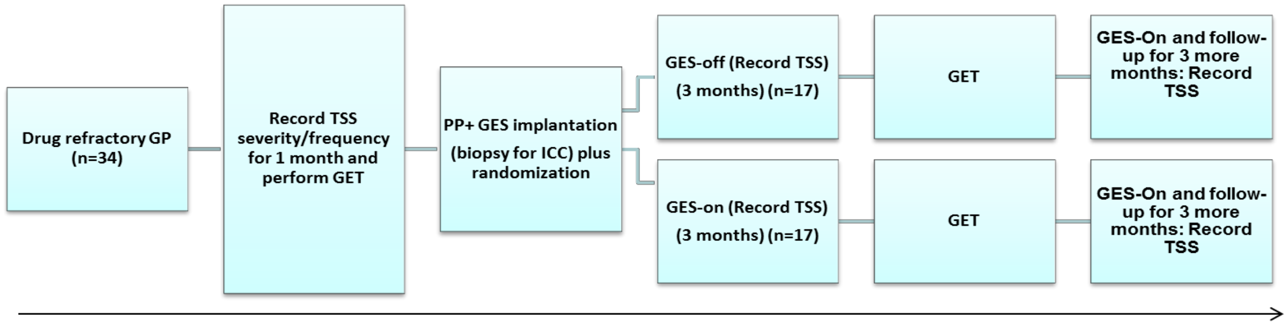

Early termination of the study will occur if a patient decides not to continue the study or if in the GES-OFF group, the nausea/vomiting symptoms are not manageable after 6 weeks of the operation, which is the usual time for surgical healing.

Histological evaluation of the biopsy samples: Paraffin embedded formalin fixed tissues will be cut at 3-4 microns thick and mounted on slides containing paired sections from cases and controls. Subsequently, the tissues will be deparaffinized and immunostained by BenchMark XT automated staining instrument. Briefly, the slides will through peroxidase block and antigen retrieval solutions before applying the primary antibodies (C-Kit clone YR145 from Cellmark, Rocklin, CA). Then, the slides will be washed and incubated with the secondary antibody followed by horseradish peroxidase (HPR). At the end, Chromogen will be added and the slides will be counter-stained by Hematoxylin. Pathology slides will be read by a pathologist who will be blind to the diagnosis of the patients, ICC will be counted per high power field (HPF) and the mean of ICC from examining 20 HPF will be calculated. For collagen fibrosis, the biopsies will be stained with trichome. Presence of diffusely distributed collagen between single muscle fibers will be defined as fibrosis and graded as mild, moderate and severe.

Gastric emptying test: Gastric emptying test will be performed utilizing a standardized meal consisting of egg beaters labeled with 99m technetium sulfur colloid accompanied by 2 toasts with strawberry jelly and 120 cc of water will be given to the patients (total calories- 240 and 2% fat). Anterior and posterior images will be obtained in the standing position immediately after meal ingestion and at 30 minutes, 1, 2, 3 and 4 hours. Geometric mean calculation and decay correction will be performed on all images. The radiologist will also be blind to the treatment groups.

The radiation exposure of a gastric emptying is equivalent to a chest x-ray or flying across country on an airline. Therefore, this test can be justified for repeating in 3 months.

D4) Statistical analysis: In the current study, the following parameters will be analyzed:

1) Changes of total gastroparesis symptom scores (TSS) and gastric retention of the radiolabeled meal (%) at 2-4 hrs in each group. TSS will include both severity and frequency.
2) Total Symptom Score (TSS) with severity and frequency of gastroparesis symptoms in each etiological subgroup of patients if enough study power would be achieved.
3) Associations between changes in gastric emptying and TSS.
4) Associations between changes in gastric emptying and ICC count/ pyloric fibrosis.

5) Associations between changes in TSS and ICC count/pyloric fibrosis.

6) SF-36, BDI, STAI and PHQ-15 scores and HBA1c (in diabetics) and their association with the treatment as well as gastric emptying/pathological findings.

The normal distribution of the data will be tested based on D'Agostino-Pearson omnibus test, were normality indicates parametric tests. The numerical variables will be reported as means (SD) or Median (Range) at baseline and follow-up visits and will be compared either by appropriate parametric or nan- parametric tests including t-test analysis or Mann–Whitney U test. The time-trends will be analyzed by either Repeated-Measure ANOVA or non-parametric Friedman Test followed by appropriate Post Hoc. Associations will be assessed based on Pearson correlation or Spearman's rho analysis. Data will be presented as bar graphs, time-trend graphs or scatter-plots and tables.

Amendment to the Protocol #1 Version 08-30-2018

Throughout the duration of the study additional relevant information collected from UMC study subject’s charts will include the following:

1. Gastric Emptying Tests
2. Endoscopy Reports
3. Lab Results
4. OR Report
5. Discharge notes
6. Pathology Reports

Amendment to the Protocol #2 Version 11-20-2018

This amendment is generated to incorporate the following statements criteria and clarifications related to this protocol:

1. Research personnel will contact patients throughout the duration of the project in order to answer any clinical and research-related questions. This may be unscheduled phone calls/ or conversations during visits.

2. Once patients have completed the study period of approximately 6 months, follow-ups will continue to occur under the Humanitarian Device Exemption (HDE) - Study E09056. These clinic follow-up visits will be conducted as they are described under HDE protocol and will take place as long as the subject has the system, in order to ensure the safety and feasibility of such therapy, which could last up to 10 years.

3. Due to the fact many subjects are from out of town the complication of having the follow-up appointments fall exactly at 3 and 6 months will be inevitably missed by many. Therefore we would like to include ± 6 weeks grace period in order to obtain the follow-up symptom information within a window of time adequate for all subjects enrolled.

4. We would like to increase the upper limit of age of patients who could be recruited and enrolled in this study. Therefore we are changing the age 65 to the age of 70 based on a statement provided by the manufacturer which is already mentioned in this study-specific consent form. *“Safety and Effectiveness of this system have not been established for patients under the age of 18 and over the age of 70”.*

5. We would like to exclude the Beck Depression Inventory (BDI) and State-trait Anxiety Inventory (STAI) instruments from the list of questionnaires/ assessments included originally in the protocol. As from the beginning, we are aware that they are very expensive and not relevant for this study. Those questionnaires were never submitted for IRB approval and were never obtained any answers from participating subjects.

6. We are including the following statement into the protocol:
“Due to the fact that many of our participants are diabetics and they are diagnosed with retinopathy possible legal blindness, or they have tremor/shakes created by certain drugs (Reglan), they are unable to read questions, and provide numbers or circle proper responses. In such situations that there are no family members to help with those tasks it is feasible for research study personnel to assist with reading and marking answers provided by the patient. It is very important to mention that the integrity of those answers are never influenced by the personnel. Only truthful assessments of participating patients in accordance with their perception and their evaluation of signs and symptoms of GP and quality of life are recorded. Therefore sometimes answers on PAGI-SYM, TSS, PQ, SF-36 could be marked by family members or research personnel.”

7. It is feasible to obtain an assessment of study-related questions via phone conversation with the patient if for any reason (long distance, personal/family issues) they are not able to come for a clinical visit as it is described in the protocol. All answers and assessments are captured precisely the way they are expressed by the patient in order to protect the integrity of the study and clinical outcomes.

8. Page 9, first paragraph of the originally approved protocol has the following statement ”*Early termination of the study will occur if a patient decides not to continue the study or if in the GES-OFF group, the nausea/vomiting symptoms are not, manageable after 6 weeks of the surgery…”* We would like to clarify that this termination in the GES-OFF group is describing only the shortening, limitation, and cutting off of the duration of the first double-blind phase of the study. This step is not finalizing participation in a clinical trial at large; it only prematurely ends the post-surgical ON or OFF phase. This could happen when GP symptoms are not

being controlled well, and are not acceptable by participating patient, regardless of the status of GES stimulation pattern, which follows precisely the randomization code. Further follow-up visits are expected in the next 3 months; therefore, the patient is going to continue participation in our clinical trial as it was proposed by the study design.

References:

1. Parkman HP, McCallum RW. Gastroparesis: pathophysiology, presentation, and treatment. New York: Humana Press, 2012.
2. Hasler WL. Symptomatic management for gastroparesis: antiemetics, analgesics, and symptom modulators. Gastroenterol Clin North Am 2015;44:113-26.
3. Sarosiek I, Davis B, Eichler E, et al. Surgical approaches to the treatment of gastroparesis: gastric electrical stimulation, pyloroplasty, total gastrectomy and enteral feeding tubes. Gastroenterol Clin North Am 2015;44:151-67.
4. Sarosiek I, Forster J, Lin Z, et al. The addition of pyloroplasty as a new surgical approach to enhance the effectiveness of gastric electrical stimulation therapy in patients with gastroparesis. Neurogastroenterol Motil 2013;25:134-e80.
5. Bashashati M, McCallum RW. Is Interstitial Cells of Cajalopathy Present in Gastroparesis? 2015;21:486-93.
6. McCallum RW, Lin Z, Forster J, et al. Gastric electrical stimulation improves outcomes of patients with gastroparesis for up to 10 years. Clin Gastroenterol Hepatol 2011;9
