# Supplementary material for: Combined Gastric Electrical Stimulation and Pyloroplasty in Gastroparesis: A Randomized Clinical Trial
Source: JAMA Netw Open. 2025 Dec 9;8(12):e2546332. doi: 10.1001/jamanetworkopen.2025.46332 (PMC12690432; doi:10.1001/jamanetworkopen.2025.46332)
Supplement: Supplement 2. — eMethods eTable 1. Median Changes in Outcome Measures at 3 Months in PP+GES-ON and PP+GES-OFF Groups Using the Wilcoxon Rank Sum Test eTable 2. Unadjusted and Adjusted Differences in Improvement in Primary Outcomes at 3 Months Between the PP+GES-ON and PP+GES-OFF Groups Using Median Regressions eTable 3. Comparisons of Mean Changes in Outcome Measures From Baseline at 3 Months Between the PP+GES-ON and PP+GES-OFF Groups eTable 4. The Trend in the Outcome Measures in the PP+GES-ON Group eTable 5. Effects of PP Alone and PP+GES on the Outcome Measures in the PP+GES-OFF Group eTable 6. Median Changes in Outcome Measures at 6 Months in PP+GES-ON and PP+GES-OFF Groups eTable 7. Comparison of Mean Changes in Outcome Measures at 6 Months Between PP+GES-ON and PP+GES-OFF Groups eFigure. Median GCSI Subscale Scores Representing PP+ GES-ON vs PP+GES-OFF Groups eReferences [file jamanetwopen-e2546332-s002.pdf]

Supplemental Online Content

Sarosiek I, Bashashati M, Davis BR, et al. Combined gastric electrical stimulation and pyloroplasty in gastroparesis: a randomized clinical trial. *JAMA Netw Open*. 2025;8(12):e2546332. doi:10.1001/jamanetworkopen.2025.46332

eMethods
eTable 1. Median Changes in Outcome Measures at 3 Months in PP+GES-ON and PP+GES-OFF Groups Using the Wilcoxon Rank Sum Test
eTable 2. Unadjusted and Adjusted Differences in Improvement in Primary Outcomes at 3 Months Between the PP+GES-ON and PP+GES-OFF Groups Using Median Regressions
eTable 3. Comparisons of Mean Changes in Outcome Measures From Baseline at 3 Months Between the PP+GES-ON and PP+GES-OFF Groups
eTable 4. The Trend in the Outcome Measures in the PP+GES-ON Group
eTable 5. Effects of PP Alone and PP+GES on the Outcome Measures in the PP+GES-OFF Group
eTable 6. Median Changes in Outcome Measures at 6 Months in PP+GES-ON and PP+GES-OFF Groups
eTable 7. Comparison of Mean Changes in Outcome Measures at 6 Months Between PP + GES-ON and PP + GES-OFF Groups
eFigure. Median GCSI Subscale Scores Representing PP+ GES-ON vs PP+GES-OFF Groups
eReferences

This supplemental material has been provided by the authors to give readers additional information about their work.

© 2025 Sarosiek I et al. *JAMA Network Open*.

eMethods.

Study-specific criteria for enrollment- other conditions: Patients who required regular MRI for their other medical diagnoses (e.g., pituitary adenoma) or had a positive pregnancy test were excluded. Peritoneal dialysis was also a contraindication for GES implantation, while hemodialysis was not. Intermittent marijuana usage was acceptable for alleviating severe nausea and enhancing appetite. Tramadol was permissible for the treatment of musculoskeletal pain when administered at a rate of no more than two times per week.

Gastric Emptying test (GE): GE tests were conducted at baseline and 3 months follow-up using standardized scintigraphy.[1] Before the test, prokinetic agents were discontinued for at least 3 days, but antiemetics were permitted to prevent vomiting during the test. Glucose was monitored before the study and had to be <275 mg/dl for the patient to be qualified for the test.

Surgical Technique: Patients simultaneously underwent a Heineke-Mikulicz pyloroplasty and GES implantation by laparotomy or robot-assisted approaches[2-4]. The Enterra GES System is comprised of: (1) a battery-powered implantable pulse-generator (Medtronic Enterra Therapy Model 3116 or Model 37800), (2) two 35-cm-long leads with 1-cm intramuscular electrodes on the ends (Model 4351; Medtronic). They were implanted in the gastric smooth muscle 9 and 10 cm from the pylorus on the greater curvature of the stomach. An external programmer interrogates the pulse generator and provides information for the adjustment of electrical parameters utilizing the principle that Voltage (V) is contingent upon the measurements of impedance (R); as well as Ohm's Law, which states that voltage equals resistance multiplied by current (I) ($V = IR$). Intraoperative

© 2025 Sarosiek I et al. *JAMA Network Open*.

endoscopy was utilized to rule out leaks, assess for patency of the pyloric channel, and rule out accidental penetration of the implanted electrodes into the stomach.[5,6] Patients received prophylactic intravenous antibiotics before and for one day postoperatively. Routine post-operative abdominal radiographs were taken to demonstrate the initial position of the electrodes and GES device.

Randomization process and programming of the GES system: The GES was initially interrogated but not activated in the operating room to confirm the integrity of the System before the surgery was completed. Randomization was performed by an unblinded physician, who conducted the activation of GES before each patient was discharged from the hospital. This step ensured that all patients had interaction with that investigator, although they were unaware of whether their devices were turned ON or OFF. The default parameters for patients randomly allocated to the PP+GES-ON group were established by the block randomization code, which was personally created and maintained only by the unblinded investigator.

At the outset, GES was set to default parameters: specifically, a pulse width of 330 μs (microseconds), current of 5 mA, a rate of 14 Hz, with a cycle ON of 0.1 seconds and a cycle OFF of 5.0 seconds. The GES was not further interrogated during these 3 months of the double-blinded phase of the study. The GES was not activated for patients assigned to the PP+GES-OFF group during the initial 3 months, but their devices were then turned ON to the default settings for the subsequent 3 months of the open-label phase of the study. The second 3-month period was open-label, so patients knew their stimulator was turned ON.

Assessment of clinical outcomes: The TSS was calculated by summing the severity of

© 2025 Sarosiek I et al. *JAMA Network Open*.

six gastroparesis symptoms: nausea (N), vomiting (V), early satiety (ES), bloating (B), post-prandial fullness (PPF), and abdominal pain (AP). Each symptom was rated on a scale from 0 to 4, where 0 indicates the absence and 4 designates extremely severe symptoms requiring bed rest and frequent emergency room visits or hospitalization. The maximum score for TSS is 24 points (higher scores indicate worse symptoms).

The PAGI-SYM is a self-administered tool designed to assess the intensity of symptoms in patients with upper gastrointestinal functional disorders, specifically gastroparesis. The GCSI, a component of PAGI-SYM, focuses on the nine most significant aspects of gastroparesis. It is derived from the average of three subscales: 1) nausea/vomiting, retching 2) post-prandial fullness, inability to finish a meal, excessive fullness, and loss of appetite, and 3) bloating and abdominal distention. Each subscale is computed by averaging 2-4 items on a 6-point Likert scale (0 indicating none and 5 indicating very severe), measuring symptom severity. [7]

Changes in symptom scores from baseline (Δ changes) were compared at 3 months and 6 months within and between the PP+GES-ON and PP+GES-OFF groups. Hemoglobin A1c (HbA1c) changes were also assessed among DMGP patients.

Hospital length of stay (HLOS) was measured as the mean number of days patients were hospitalized within each 3-month follow-up period, compared with baseline. The baseline HLOS was calculated using data from the 12 months preceding the surgery, divided into four quarters. The hospitalization period for the surgery itself was excluded from this measurement. Only hospitalizations due to exacerbations of gastroparesis symptoms (e.g., vomiting associated with abdominal pain, diabetic ketoacidosis (DKA),

© 2025 Sarosiek I et al. *JAMA Network Open*.

hypoglycemia) or infections affecting glucose control were included. Participants were instructed to promptly notify staff of any health concerns requiring medical attention or hospitalization. Documentation of the events was made at the primary study-related visits, which were 3 months after randomization, as well as during any visits necessitated by patient complaints and health concerns. The visits were overseen and supervised by a blinded clinician and research coordinators who were unaware of the details of the randomization code. Serious and non-serious adverse events (SAEs and AEs) were reviewed, documented, followed up, and submitted for IRB acknowledgment if they met the SAE definition.

Sample size: To address symptom improvement, which was the primary outcome measure of this study, we calculated the sample size based on symptom scores as outlined in the main text. We also calculated the sample size based on the GE data as outlined below. For the GE, after considering that pyloroplasty is not inferior to GES plus pyloroplasty, the sample size for the non-inferiority trial, including % normalization of the GE as the binary outcome, was calculated. GE was normalized in 60 % of gastroparesis patients with GES-ON plus Pyloroplasty based on our database. On the other hand, GES alone normalizes GE in around 18% of gastroparesis patients, predominantly in idiopathic gastroparesis. By defining the non-inferiority limit equal to (60 - 18= 42%), and the percentage of success in the GES-ON plus pyloroplasty group, 17 patients had to be included in each arm of this study. Therefore, selecting 18 subjects per group, based on the symptom score criteria described in the main text, would provide sufficient power to compare gastric emptying as well.

Statistical analysis: Data were analyzed using Stata 17 version (StataCorp LLC, College Station, TX). Considering the Likert scale response data, we primarily analyzed all data

© 2025 Sarosiek I et al. *JAMA Network Open*.

with nonparametric analyses and further validated them by corresponding parametric analyses.

eTable 1. Median Changes in Outcome Measures at 3 Months in PP+GES-ON and PP+GES-OFF Groups Using the Wilcoxon Rank Sum Test

Factor	**PP + GES-ON (N=19)**	**PP + GES-OFF (N=19)**	**p-value**
	**Median change from baseline (Q1, Q3)**	**Median change from baseline (Q1, Q3)**	
**TSS**	-15.0 (-16.0, -8.0)	-3.0 (-10.0, -1.0)	0.005
Vomiting score	-2.0 (-3.0, -2.0)	0.0 (-2.0, 0.0)	0.008
Nausea score	-2.0 (-3.0, -1.0)	-1.0 (-2.0, 0.0)	0.037
Early satiety score	-2.0 (-3.0, -1.0)	-1.0 (-2.0, 0.0)	0.069
Bloating score	-2.0 (-3.0, -1.0)	0.0 (-2.0, 0.0)	0.042
Postprandial fullness score	-2.0 (-3.0, -1.0)	-1.0 (-2.0, 0.0)	0.084
Epigastric pain	-3.0 (-3.0, -2.0)	0.0 (-2.0, 0.0)	0.005
GCSI-total	-2.2 (-2.6, -1.5)	-0.9 (-1.8, -0.4)	0.015
GCSI-nausea/vomiting	-2.3 (-3.3, -1.3)	-1.0 (-2.7, -0.3)	0.021
GCSI-postprandial fullness	-2.2 (-2.5, -1.0)	-1.0 (-2.0, 0.2)	0.10
GCSI-bloating	-2.0 (-3.5, 0.0)	-1.0 (-2.0, 0.5)	0.18
**PAGI-SYM**	-2.1 (-2.8, -1.3)	-0.8 (-1.6, -0.1)	0.009
**HbA1c in DM**	-1.0 (-1.2, -0.3)	0.1 (-0.6, 1.0)	0.093
**Hospitalization days**	0.0 (-9.0, 0.0)	-1.5 (-7.2, 0.0)	0.95
**Hospitalizations, N(%)**	6 (32%)	7 (37%)	0.73
**GE at 2 hr†**	-18.0 (-42.0, -1.5)	-26.0 (-43.0, -10.0)	0.61
**GE at 4 hr†**	-30.0 (-42.0, -11.0)	-17.0 (-47.0, -6.0)	0.54

© 2025 Sarosiek I et al. *JAMA Network Open*.

Q1, Q3: Interquartile Range (IQR); DM: Diabetes Mellitus; GP: Gastroparesis; GE: Gastric Emptying; TSS: Total Symptom Scores; GCSI: Gastroparesis Cardinal Symptom Index; PAGI-SYM: Patient Assessment of Upper Gastrointestinal Symptom Severity Index; PP+GES-ON: OFF. Pyloroplasty (PP) with Gastric Electrical Stimulation (GES)-ON; PP+GES-OFF: Pyloroplasty (PP) with Gastric Electrical Stimulation (GES); p-values were computed by chi-square for categorical variables and the Wilcoxon rank sum for continuous variables; † (% retention).

eTable 2. Unadjusted and Adjusted Differences in Improvement in Primary Outcomes at 3 Months Between the PP+GES-ON and PP+GES-OFF Groups Using Median Regressions (N=38)

	Unadjusted analysis				Adjusted analysis*			
	MD	95%CI		p-value	MD	95%CI		p-value
TSS	-12.00	-17.49	-6.51	<0.001	-12.00	-18.02	-5.98	<0.001
GCSI	-1.33	-2.34	-0.33	0.01	-1.36	-2.31	-0.41	0.006
PAGI-SYM	-1.25	-2.17	-0.33	0.009	-1.21	-2.10	-0.33	0.009

MD: median difference; CI: confidence interval; TSS: Total Symptom Scores; GCSI: Gastroparesis Cardinal Symptom Index; PAGI-SYM: Patient Assessment of Upper Gastrointestinal Symptom Severity Index; PP+GES-ON: Pyloroplasty (PP) with Gastric Electrical Stimulation (GES)-ON; PP+GES-OFF: Pyloroplasty (PP) with Gastric Electrical Stimulation (GES)-OFF; *Adjusted analysis included GP type and gastric emptying at 2 hr.

© 2025 Sarosiek I et al. *JAMA Network Open*.

eTable 3. Comparisons of Mean Changes in Outcome Measures From Baseline at 3 Months Between the PP+GES-ON and PP+GES-OFF Groups

Outcomes	PP + GES-ON (N=17)	PP + GES-OFF (N=18)	Mean difference between ON and OFF groups	95%Confidnec interval		p-value
	Mean change from baseline (SD)	Mean change from baseline (SD)				
**TSS**	-11.9 (6.1)	-5.6 (7.2)	-6.33	-10.93	-1.73	0.008
Vomiting score	-2.2 (1.4)	-0.8 (1.7)	-1.40	-2.47	-0.33	0.01
Nausea score	-2.0 (1.1)	-1.1 (1.7)	-0.94	-1.91	0.02	0.06
Early satiety score	-1.9 (1.6)	-0.8 (1.7)	-1.11	-2.23	0.01	0.05
Bloating score	-1.7 (1.2)	-0.9 (1.5)	-0.82	-1.77	0.14	0.09
Postprandial fullness score	-1.8 (1.5)	-0.9 (1.3)	-0.88	-1.86	0.10	0.08
Epigastric pain	-2.1 (1.3)	-0.8 (1.4)	-1.34	-2.26	-0.42	0.006
GCSI-total	-2.1 (1.2)	-1.2 (1.5)	-0.88	-1.80	0.88	0.06
GCSI-nausea/vomiting	-2.5 (1.3)	-1.4 (1.8)	-1.06	-2.14	1.06	0.05
GCSI-postprandial fullness	-2.0 (1.3)	-1.3 (1.8)	-0.69	-1.76	0.69	0.20
GCSI-bloating	-1.9 (1.7)	-1.0 (2.0)	-0.88	-2.19	0.88	0.18
**PAGI-SYM**	-2.0 (1.0)	-1.2 (1.3)	-0.82	-1.65	0.82	0.05
**HbA1c in DM**	-1.1 (1.2)	-0.1 (2.1)	-1.04	-2.77	0.68	0.22
**Hospitalization days**	-3.9(5.7)	-3.0(7.7)	-0.89	-5.76	3.99	0.71
**GE at 2 hr†**	-21.9 (29.2)	-25.7 (22.2)	3.80	-15.36	22.95	0.69
**GE at 4 hr†**	-22.3 (33.0)	-22.5 (24.7)	0.15	-21.38	21.69	0.99

© 2025 Sarosiek I et al. *JAMA Network Open*.

SD: Standard Deviation; DM: Diabetes Mellitus; GP: Gastroparesis; GE: Gastric Emptying; TSS: Total Symptom Scores; GCSI: Gastroparesis Cardinal Symptom Index; PAGI-SYM: Patient Assessment of Upper Gastrointestinal Symptom Severity Index; PP+GES-ON: Pyloroplasty (PP) with Gastric Electrical Stimulation (GES)-ON; PP+GES-OFF: Pyloroplasty (PP) with Gastric Electrical Stimulation (GES)-OFF; p-values were computed by unpaired t-tests; † (% retention).

eTable 4. The Trend in the Outcome Measures in the PP+GES-ON Group

Factor	**Baseline**	**3-month**	**6-month**	**Percent improvement**				
	**Mean (SD)**	**Mean (SD)**	**Mean (SD)**	**% Mean change (SD) at 3m from baseline**	**% Mean change (SD) at 6m from baseline**	**Baseline vs. 3 months, p-value**	**Baseline vs. 6 months, p-value**	**3 months vs. 6 months, p-value**
**TSS**	19.7 (2.8)	7.8 (5.1)	8.6 (6.8)	59.3 (28.3)	55.5 (36.1)	<0.001	<0.001	1.00
Vomiting score	3.2 (0.8)	0.9 (1.1)	1.5 (1.3)	67.2 (42.5)	48.9 (53.5)	<0.001	<0.001	0.49
Nausea score	3.7 (0.6)	1.7 (1.0)	1.6 (1.3)	53.9 (28.3)	55.0 (41.8)	<0.001	<0.001	1.00
Early satiety score	3.4 (0.6)	1.5 (1.2)	2.1 (1.4)	52.0 (44.7)	34.4 (45.0)	<0.001	0.005	0.23
Bloating score	2.8 (0.9)	1.1 (1.1)	1.4 (1.2)	58.8 (40.1)	51.7 (44.5)	<0.001	<0.001	1.00
Postprandial fullness score	3.4 (0.6)	1.6 (1.2)	1.9 (1.0)	51.0 (41.7)	42.2 (28.9)	<0.001	0.001	0.63
Epigastric pain	3.2 (0.6)	1.1 (1.2)	1.3 (1.4)	67.6 (41.2)	58.3 (44.4)	<0.001	<0.001	1.00
**GCSI-total**	3.8 (0.8)	1.6 (1.2)	1.8 (1.2)	58.4 (30.0)	49.3 (33.2)	<0.001	<0.001	0.59
GCSI-nausea/vomiting	3.8 (1.1)	1.2 (1.1)	1.8 (1.5)	66.3 (25.0)	51.3 (40.3)	<0.001	<0.001	0.27
GCSI-postprandial fullness	4.0 (0.7)	2.0 (1.2)	2.3 (1.4)	49.5 (33.5)	41.4 (38.4)	<0.001	<0.001	1.00
GCSI-bloating	3.5 (1.1)	1.5 (1.8)	1.5 (1.4)	57.9 (48.4)	55.8 (37.6)	<0.001	<0.001	1.00
**PAGI-SYM**	3.5 (0.8)	1.4 (0.9)	1.5 (1.0)	59.6 (26.3)	53.7 (31.2)	<0.001	<0.001	1.00
**HbA1c in DM**	8.3 (2.3)	6.8 (1.4)	6.6 (1.7)	12.9 (11.7)	21.3 (7.6)	0.05	0.15	1.00
**Hospitalization days**	5.2 (6.7)	0.9 (1.8)	0.8 (1.7)	77.7 (39.3)	56.4 (87.8)	0.015	0.12	1.00
**GE at 2 hr†**	78.5 (17.6)	54.6 (25.3)		24.6 (40.4)		0.009*		
**GE at 4 hr†**	48.3 (23.5)	20.4 (23.5)		30.5 (124.4)		0.02*		

SD: Standard Deviation; DM: Diabetes Mellitus; GP: Gastroparesis; GE: Gastric Emptying; TSS: Total Symptom Scores; GCSI: Gastroparesis Cardinal Symptom Index; PAGI-SYM: Patient Assessment of Upper Gastrointestinal Symptom Severity Index; PP+GES-ON: Pyloroplasty (PP) with Gastric Electrical

© 2025 Sarosiek I et al. *JAMA Network Open*.

Stimulation (GES)-ON.P-values were computed by repeated measures analysis of variance followed by post hoc multiple comparisons after adjusting for Bonferroni's correction; * p-values were calculated by paired t-tests; † % retention.

eTable 5. Effects of PP Alone and PP+GES on the Outcome Measures in the PP+GES-OFF Group

Factor	Baseline	3-month	6-month	Percent improvement		(PP effect) Baseline vs. 3 months, p-value	(PP+GES effect) Baseline vs. 6 months, p-value	(GES effect) 3 months vs. 6 months, p-value
	Mean (SD)	Mean (SD)	Mean (SD)	% Mean change (SD) at 3m from baseline	% Mean change (SD) at 6m from baseline			
**TSS**	17.9 (3.6)	12.3 (6.6)	7.3 (5.9)	29.8 (34.8)	58.9 (31.8)	0.002	<0.001	0.006
Vomiting score	3.2 (0.8)	2.4 (1.5)	1.3 (1.3)	20.8 (51.9)	56.9 (47.1)	0.07	<0.001	0.02
Nausea score	3.4 (0.7)	2.3 (1.3)	1.2 (1.1)	25.0 (50.6)	60.3 (41.2)	0.02	<0.001	0.02
Early satiety score	3.0 (1.0)	2.2 (1.4)	1.1 (1.3)	27.9 (52.0)	64.1 (43.2)	0.08	<0.001	0.03
Bloating score	2.8 (1.1)	1.9 (1.6)	1.3 (1.2)	32.8 (49.7)	56.8 (36.3)	0.02	<0.001	0.12
Postprandial fullness score	2.7 (1.3)	1.8 (1.4)	1.4 (1.1)	40.1 (41.1)	55.0 (35.5)	0.02	<0.001	0.46
Epigastric pain	2.5 (1.4)	1.7 (1.5)	1.0 (1.4)	37.8 (43.4)	64.4 (45.0)	0.08	<0.001	0.06
**GCSI-total**	3.5 (0.9)	2.3 (1.2)	1.5 (1.2)	32.3 (33.4)	54.6 (37.2)	0.001	<0.001	0.07
GCSI-nausea/vomiting	3.8 (1.2)	2.4 (1.4)	1.4 (1.1)	29.3 (42.9)	56.6 (32.6)	0.001	<0.001	0.06
GCSI-postprandial fullness	3.4 (1.2)	2.1 (1.4)	1.6 (1.5)	29.3 (48.4)	44.0 (51.1)	0.005	<0.001	0.59
GCSI-bloating	3.3 (1.8)	2.2 (1.5)	1.4 (1.4)	18.0 (76.6)	40.6 (69.7)	0.08	0.001	0.22
**PAGI-SYM**	3.0 (1.0)	1.8 (1.0)	1.2 (0.9)	35.9 (33.4)	57.7 (31.8)	<0.001	<0.001	0.11
**HbA1c in DM**	8.9 (2.1)	9.5 (2.4)	7.6 (1.2)	0.1 (20.5)	15.7 (11.8)	1.00	0.31	0.54
**Hospitalization days**	6.1 (6.3)	3.1 (7.8)	1.2 (2.2)	49.8 (67.3)	68.6 (63.2)	0.18	0.06	1.00
**GE at 2 hr†**	69.0 (17.6)	41.5 (25.0)		38.6 (31.5)		0.001*		

© 2025 Sarosiek I et al. *JAMA Network Open*.

**GE at 4 hr†**	44.0 (25.8)	21.1 (24.9)		52.2 (38.2)		0.003*		

Q1, Q3: Interquartile Range; SD: Standard Deviation; DM: Diabetes Mellitus; GP: Gastroparesis; GE: Gastric Emptying; TSS: Total Symptom Scores; GCSI: Gastroparesis Cardinal Symptom Index; PAGI-SYM: Patient Assessment of Upper Gastrointestinal Symptom Severity Index; PP+GES-OFF: Pyloroplasty (PP) with Gastric Electrical Stimulation (GES)-OFF; P-values were computed by repeated measures analysis of variance followed by post hoc multiple comparisons after adjusting for Bonferroni's correction; *p-values are calculated by paired t-tests; † %retention.

eTable 6. Median Changes in Outcome Measures at 6 Months in PP+GES-ON and PP+GES-OFF Groups

Factor	**PP + GES-ON (N=17)**	**PP + GES-OFF (N=18)**	**p-value**
N	**Median (Q1, Q3)**	**Median (Q1, Q3)**	
**TSS**	-11.0 (-17.0, -6.0)	-11.0 (-14.0, -6.0)	0.77
Vomiting score	-2.0 (-2.0, -1.0)	-2.0 (-3.0, -1.0)	0.44
Nausea score	-2.0 (-3.0, -2.0)	-2.0 (-3.0, -1.0)	0.88
Early satiety score	-1.0 (-3.0, 0.0)	-2.0 (-3.0, 0.0)	0.26
Bloating score	-2.0 (-2.0, -1.0)	-2.0 (-2.0, -1.0)	0.89
Postprandial fullness score	-1.0 (-3.0, -1.0)	-2.0 (-2.0, -1.0)	0.94
Epigastric pain	-2.0 (-3.0, -1.0)	-2.0 (-3.0, 0.0)	0.67
GCSI-total	-1.5 (-2.6, -1.0)	-1.6 (-2.9, -0.8)	0.71
GCSI-nausea/vomiting	-1.7 (-3.2, -0.3)	-2.3 (-3.3, -1.0)	0.34
GCSI-postprandial fullness	-2.1 (-2.8, -0.8)	-1.8 (-3.8, 0.0)	0.97
GCSI-bloating	-1.8 (-2.8, -0.8)	-2.0 (-3.5, 0.0)	0.97
**PAGI-SYM**	-1.7 (-2.4, -1.0)	-1.7 (-2.5, -1.0)	0.93
**HbA1c in DM**	-1.3 (-3.6, -1.0)	-1.6 (-2.4, -0.7)	0.80
**Hospitalization days**	0.0 (-6.3, 0.0)	-3.8 (-9.1, 0.0)	0.40
**Hospitalizations, N(%)**	2(22%)	4(29%)	0.74

Q1, Q3: Interquartile Range; DM: Diabetes Mellitus; GP: Gastroparesis; GE: Gastric Emptying; TSS: Total Symptom Scores; GCSI: Gastroparesis Cardinal Symptom Index; PAGI-SYM: Patient Assessment of Upper Gastrointestinal Symptom Severity Index; PP+GES-ON: OFF. Pyloroplasty (PP) with Gastric Electrical Stimulation (GES)-ON; PP+GES-OFF: Pyloroplasty (PP) with Gastric Electrical Stimulation (GES); p-values were computed by chi-square for categorical

© 2025 Sarosiek I et al. *JAMA Network Open*.

variables and the Wilcoxon rank sum for continuous variables; sample size per group was different for HbA1c and hospitalizations.

eTable 7. Comparison of Mean Changes in Outcome Measures at 6 Months Between PP + GES-ON and PP + GES-OFF Groups

Factor	**PP + GES-ON (N=17)**	**PP + GES-OFF (N=18)**	
	**Mean (SD)**	**Mean (SD)**	**p-value**
**TSS**	-11.0 (7.2)	-10.6 (6.5)	0.86
Vomiting score	-1.6 (1.5)	-1.9 (1.5)	0.60
Nausea score	-2.1 (1.5)	-2.2 (1.4)	0.83
Early satiety score	-1.3 (1.6)	-1.9 (1.8)	0.32
Bloating score	-1.5 (1.2)	-1.6 (1.2)	0.78
Postprandial fullness score	-1.5 (1.1)	-1.4 (1.4)	0.91
Epigastric pain	-1.9 (1.6)	-1.6 (1.5)	0.61
GCSI-total	-1.8 (1.2)	-2.0 (1.6)	0.62
GCSI-nausea/vomiting	-1.9 (1.6)	-2.4 (1.6)	0.34
GCSI-postprandial fullness	-1.7 (1.5)	-1.8 (1.9)	0.78
GCSI-bloating	-1.8 (1.4)	-1.9 (2.3)	0.95
**PAGI-SYM**	-1.8 (1.1)	-1.8 (1.3)	0.95
**HbA1c in DM**	-2.0 (1.4)	-1.6 (1.3)	0.71
**Hospitalization days**	-3.3 (6.2)	-5.5 (6.5)	0.45

SD: Standard Deviation; DM: Diabetes Mellitus; GP: Gastroparesis; GE: Gastric Emptying; TSS: Total Symptom Scores; GCSI: Gastroparesis Cardinal Symptom Index; PAGI-SYM: Patient Assessment of Upper Gastrointestinal Symptom Severity Index; PP+GES-ON: Pyloroplasty (PP) with Gastric Electrical

© 2025 Sarosiek I et al. *JAMA Network Open*.

Stimulation (GES)-ON; PP+GES-OFF: Pyloroplasty (PP) with Gastric Electrical Stimulation (GES)-OFF; p-values were computed by unpaired t-tests; sample size per group was different for HbA1c and hospitalizations.

eFigure. Median GCSI Subscale Scores Representing PP+ GES-ON vs PP+GES-OFF Groups

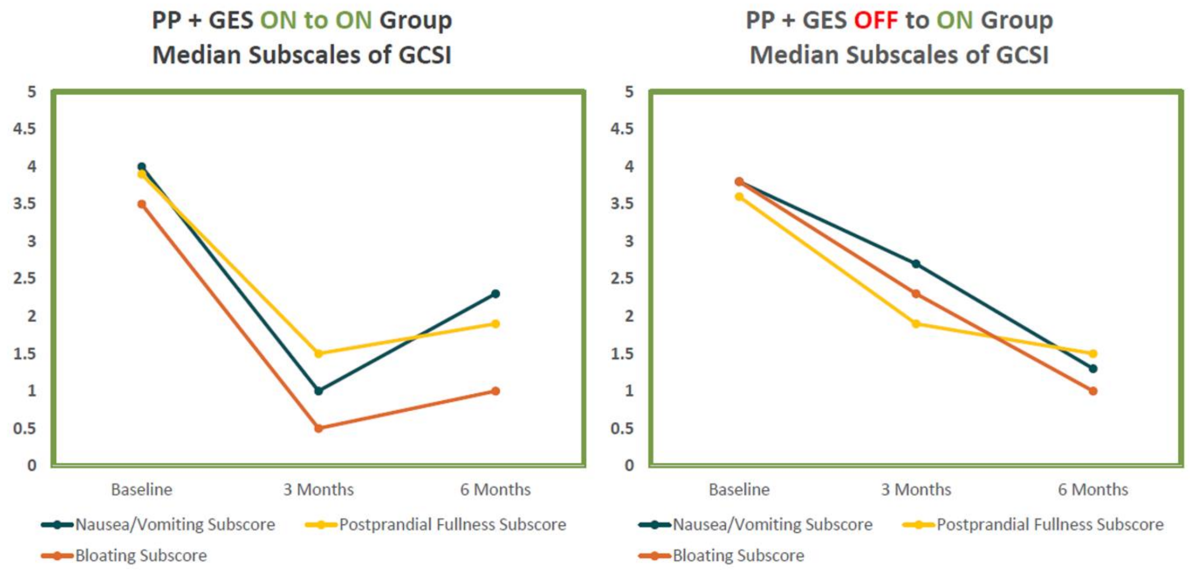

At 3 months, the median GCSI subscale score for nausea/vomiting was significantly lower in ON vs. OFF group (p=0.03).

COMPLICATIONS AND SAEs: No immediate postoperative problems or major adverse events were observed following the tandem GES and PP surgeries. Specifically, there were no instances of visceral perforation, anastomosis leak, or surgical site infection.

© 2025 Sarosiek I et al. *JAMA Network Open*.

Additionally, no technical difficulties related to the positioning or implantation of the electrodes, placement of the pulse generator, interrogation of parameters, or malfunctioning of the device were encountered.

eReferences

1. Abell TL, Camilleri M, Donohoe K, et al. Consensus recommendations for gastric emptying scintigraphy: a joint report of the American Neurogastroenterology and Motility Society and the Society of Nuclear Medicine. *J Nucl Med Technol*. Mar 2008;36(1):44-54. doi:10.2967/jnmt.107.048116
2. Heineke. Operation de Pylorusstenose. Inaug. Dissert., Furth. 1886.
3. Mikulicz J. Zur operativen Behandlung des stenosirenden Magengeschivurs. *Verh Deutsch Ges Chir*. 1887;16:337.
4. Davis BR, McCallum RW. Chapter 31 - Surgical management of gastroparesis. In: McCallum RW, Parkman HP, eds. *Gastroparesis*. Academic Press; 2021:431-439.
5. Abell T, McCallum R, Hocking M, et al. Gastric electrical stimulation for medically refractory gastroparesis. *Gastroenterology*. Aug 2003;125(2):421-8. doi:10.1016/s0016-5085(03)00878-3
6. Sarosiek I, McCallum R. Chapter 30 - Gastric electrical stimulation for gastroparesis. In: McCallum RW, Parkman HP, eds. *Gastroparesis*. Academic Press; 2021:413-429.
7. Revicki DA, Rentz AM, Tack J, et al. Responsiveness and interpretation of a symptom severity index specific to upper gastrointestinal disorders. *Clinical Gastroenterology and Hepatology*. 2004;2(9):769-777. doi:10.1016/S1542-3565(04)00348-9

© 2025 Sarosiek I et al. *JAMA Network Open*.
