# Supplementary material for: Combined Gastric Electrical Stimulation and Pyloroplasty in Gastroparesis: A Randomized Clinical Trial
Source: JAMA Netw Open. 2025 Dec 9;8(12):e2546332. doi: 10.1001/jamanetworkopen.2025.46332 (PMC12690432; doi:10.1001/jamanetworkopen.2025.46332)
Supplement: Supplement 3. — Data Sharing Statement [file jamanetwopen-e2546332-s003.pdf]

Data Sharing Statement

Sarosiek. Combined Gastric Electrical Stimulation and Pyloroplasty in Gastroparesis. *JAMA Netw Open*. Published December 03, 2025. doi:10.1001/jamanetworkopen.2025.46332

Data
Additional Information: https://clinicaltrials.gov/study/NCT03123809?locStr=El%20Paso,%20TX&country=United%20States&state=Texas&city=El%20Paso&cond=Gastroparesis&rank=7
Data available: Yes
Data types: Deidentified participant data
How to access data: NA
When available: With publication

Supporting Documents
Document types: None

Additional Information
Who can access the data: Approved by the IRB of the data origin institution
Types of analyses: NA
Mechanisms of data availability: signed data access agreement
